# Systematic Functional Analysis of Sigma (σ) Factors in the Phytopathogen *Xanthomonas campestris* Reveals Novel Roles in the Regulation of Virulence and Viability

**DOI:** 10.3389/fmicb.2018.01749

**Published:** 2018-08-03

**Authors:** Li-Yan Yang, Li-Chao Yang, Yong-Liang Gan, Lin Wang, Wan-Zong Zhao, Yong-Qiang He, Wei Jiang, Bo-Le Jiang, Ji-Liang Tang

**Affiliations:** ^1^State Key Laboratory for Conservation and Utilization of Subtropical Agro-Bioresources, College of Life Science and Technology, Guangxi University, Nanning, China; ^2^Guangxi Key Laboratory of Power System Optimization and Energy Technology, Guangxi University, Nanning, China

**Keywords:** sigma (σ) factor, housekeeping gene, virulence, hypersensitive response, type III secretion, *Xanthomonas*

## Abstract

The black rot pathogen *Xanthomonas campestris* pv. *campestris* (*Xcc*) is a model organism for the study of plant bacterial pathogenesis mechanisms. In bacteria, σ factors serve as important regulatory elements that respond to various environmental signals and cues. Though *Xcc* encodes 15 putative σ factors little is known about their roles. As an approach to identify the potential role of each σ factor, we constructed mutations in each of the σ-factor genes as well as generating mutants deficient in multiple σ factors to assess these regulators potential additive functions. The work identified two σ^70^ factors essential for growth. Furthermore, the work discovered a third σ^70^ factor, RpoE1, important for virulence. Further studies revealed that RpoE1 positively regulates the expression of the *hrp* gene cluster that encodes the type III secretion system (T3SS) which determines the pathogenicity and hypersensitive response of *Xcc* on plants. *In vivo* and *in vitro* studies demonstrated that RpoE1 could bind to the promoter region and promote transcription of *hrpX*, a gene encoding a key regulator of the *hrp* genes. Overall, this systematic analysis reveals important roles in *Xcc* survival and virulence for previously uncharacterized σ^70^ factors that may become important targets for disease control.

## Introduction

Bacteria have developed various mechanisms to accurately modify their behavior in response to changes in their environmental soundings to facilitate growth, survival, and infection. One important class of global regulators/signaling systems are the sigma (σ) factors which are typically master transcriptional regulators of gene expression. They are subunits of RNA polymerase (RNAP) and are required for transcription initiation, including the recognition and opening of promoters as well as the initial steps in RNA synthesis (Paget, 2015). These regulators are responsible for RNA synthesis in exponentially growing cells, with bacteria possessing multiple sigma factors that are used coordinately to regulate the expression of genes involved in diverse functions, including stress responses, iron uptake, morphological development, and chemotaxis (Saecker et al., 2011). The σ factors can be classified into two structurally and evolutionarily distinct families: the σ^54^ family and the σ^70^ family (Kazmierczak et al., 2005; Feklístov et al., 2014; Zhang and Buck, 2015; Davis et al., 2017). Most bacteria harbor a single number of σ^54^ family factors, which regulate the expression of genes involved in various cellular processes such as nitrogen metabolism, phage shock, flagellar motility, virulence, and cellular differentiation (Studholme and Buck, 2000; Studholme, 2002; Davis et al., 2017). However, many bacterial genomes encode a large number of σ^70^ family proteins, which can be subdivided into four groups: group I consists of housekeeping σ^70^ factors and groups II–IV are comprised of alternative σ^70^ factors with specialized functions. Normally, groups II and III members regulate the expression of genes involved in general stress responses, motility, and chemotaxis (Paget and Helmann, 2003). Group IV members, also known as ECF (extracytoplasmic function) σ^70^ factors and are numerically the largest group of σ^70^ family factors regulating the expression of genes involved in sensing and responding to stressful and adverse environmental conditions (Kazmierczak et al., 2005; Davis et al., 2017).

The genus *Xanthomonas* is a group of Gram-negative plant-associated bacteria that belong to the Gamma subdivision of Proteobacteria (Ryan et al., 2011; Rodriguez et al., 2012). *Xanthomonas* comprises of a large number of species and some of which include multiple pathovars (pv.) or subspecies (subsp.) (Ryan et al., 2011; Rodriguez et al., 2012). These plant pathogens cause severe crop diseases in important economic crops, such as rice, pepper, tomato, cassava, and citrus. Arguably the most well studied of xanthomonad plant pathogens is *Xanthomonas campestris* pv. *campestris* (*Xcc*) (hereafter *Xcc*), the causative agent of black rot disease in vegetable brassica crops worldwide (Mansfield et al., 2012; Vicente and Holub, 2013). This is due in part to the substantial amount of genome data now available which has added to our understanding of how *Xcc* causes disease (Ryan et al., 2011). It is thought that *Xcc* persists as epiphytes on the plant surface before they enter the plant via natural openings such as hydathodes, stomata, or wounds (Ryan et al., 2011). Once inside the plant tissue they multiply either locally in the intercellular space or colonize the xylem vessels and spread systemically within the plant to cause disease. A number of virulence determinants have been identified and characterized, such as effector proteins secreted by the type III secretion system (T3SS), extracellular polysaccharides (EPS), and extracellular enzymes (Ryan et al., 2011, 2015; Vicente and Holub, 2013; Zhou et al., 2017). The reason for the remarkable success of *Xcc* can be attributed to its large versatility and environment-driven flexible changes in its transcriptional profile.

To study the signaling systems that underpin the complex interactions between bacterial pathogens and their hosts will facilitate our understanding of the mechanisms by which the pathogens regulate stress and virulence processes during bacterial disease. In order to achieve this establishment of the specific roles of signaling elements and their contribution to the disease process is required. Here we have addressed this issue through a study of σ factors and their involvement in the virulence of *Xcc*. Despite σ factors being studied extensively in other Gram-negative bacteria, only limited work has been carried out in *Xcc* with the σ^54^ factor RpoN2 shown to be required for the regulation of motility (Yang et al., 2009) and one σ^70^ factor being demonstrated to be involved in stress response regulation (Bordes et al., 2011). Recently, it has been demonstrated that the σ^54^ factors are required for full virulence of *Xanthomonas citri* subsp. *citri* and *Xanthomonas oryzae* pv. *oryzae* (Tian et al., 2015; Gicharu et al., 2016). However, no σ factor has been shown to be involved in the virulence or pathogenesis of *Xcc* although the sequenced genomes of *Xcc* strains possess fifteen predicted σ factors, two being σ^54^ family members and the rest being σ^70^ family members (da Silva et al., 2002; Qian et al., 2005; Vorhölter et al., 2008).

Using the sequenced *Xcc* strain 8004 we attempted to characterize the 15 predicted σ factor-encoding genes (Qian et al., 2005). As an approach to identify their potential role in *Xcc*, we constructed mutations in each of these genes as well as generating collective mutations to assess their potential additive functions. These strains were tested in various *in vitro* and *in planta* assays. Mutants defective in each of 13 predicted σ factors were obtained, but mutants of two σ factor genes *XC_3806* (*rpoD*) and *XC_3843* (*rpoH*) could not be generated, which appeared essential for viability and survival. This was confirmed when an extra copy of the target gene was introduced into *Xcc* cells and expressed *in trans* allowing its chromosomal homolog to be deleted. The *in planta* assays revealed that one of the mutants had significantly reduced virulence. The mutant was generated by erasing one of the group IV σ^70^ factors (encoded by *XC_2974*), which is homologous to the RpoE of *Escherichia coli* and named RpoE1 in this work. Further studies revealed that RpoE1 positively affects the expression of the *hrp* genes that encode the T3SS via directly regulating the transcription of *hrpX*, a gene encoding one of the key *hrp* regulators. Ultimately, each sigma factor mutant was evaluated for pathogenicity but only RpoE1 appeared to be important for overall virulence under the conditions tested.

## Materials and methods

### Bacterial strains, plasmids, and growth conditions

The bacterial strains and plasmids used in this study are listed in Supplementary Table S1. *Xcc* strains were grown at 28°C in the nutrient rich medium NYG (5 g of peptone, 3 g of yeast extract and 20 g of glycerol per liter, pH 7.0) (Daniels et al., 1984) and the minimal medium XCM1 (1.0 g of (NH_4_)_2_SO_4_, 10.5 g of K_2_HPO_4_, 4.5 g of KH_2_PO_4_, 0.246 g of MgSO_4_•7H_2_O, 2.362 g of succinic acid, 0.15 g of casamino acids per liter, pH 6.6) (Jiang et al., 2013). *E. coli* strains were grown in LB medium (10 g of bactotryptone, 5 g of yeast extract, 5 g of sodium chloride, and 1 g of D-glucose per liter) at 37°C. Antibiotics were added at the following concentrations as required: Ampicillin (Amp) 100 μg/ml; Kanamycin (Kan) 25 μg/ml; Rifampicin (Rif) 50 μg/ml; Spectinomycin (Spc) 50 μg/ml; Tetracycline (Tc) 5 μg/ml for *Xcc* and 15 μg/ml for *E. coli*. Sucrose and isopropyl-β-D-thiogalactopyranoside (IPTG) were added as required at 10% (w/v) and 1 mM, respectively.

### DNA and RNA manipulations and conjugation between *Xcc* and *E. coli* strains

The total RNA of *Xcc* strains was extracted with a total-RNA extraction kit (Promega, Madison, Wisconsin, USA) and reverse transcription was performed using a cDNA synthesis kit (Takara, Dalian, China), according to the manufacturer's instructions. To assay the transcription level of the genes studied, qRT-PCR and sqRT-PCR were performed using the total RNA extracted from *Xcc* strains. The synergy brand (SYBR) green-labeled PCR fragments were amplified using the primer sets listed in Supplementary Table S2. The relative expression of genes was determined using the 2^−ΔΔCt^ method (Livak and Schmittgen, 2001). The 16S rRNA gene was used as an internal standard. All sqRT-PCR and qRT-PCR tests were performed in triplicate.

Plasmids were introduced from *E. coli* strain into *Xcc* strain by triparental conjugation using pRK2073 (Supplementary Table S1) as the helper plasmid as described by Turner et al. (1985). *Xcc* transconjugants were selected in NYG medium supplemented with appropriate antibiotics.

### Deletion mutant construction and complementation

*Xcc* gene deletion mutants were constructed by double-crossover homologous recombination using the suicide plasmid pK18mobsacB (Schäfer et al., 1994). For the construction of the *rpoE1* deletion mutant, 700 bp upstream and 791 bp downstream fragments flanking the *rpoE1* gene (i.e., *XC_2974*) were amplified from the genome of *Xcc* strain 8004 with the primer sets D2974LF/LR and D2974RF/RR (Supplementary Table S2). The fragments were cloned into the suicide plasmid pK18mobsacB, yielding pK2974D, which was transferred into strain 8004 by triparental conjugation and the transconjugants were screened on NYG plate supplemented with Rif and 10% sucrose. The obtained *rpoE1* deletion mutant was further confirmed by PCR and named Δ*rpoE1* (Supplementary Table S1). Other mutants with a deletion in individual σ factor-encoding gene were constructed by the same strategy and listed in Supplementary Table S1. The double deletion mutant of the two σ^54^ family factor-encoding genes (i.e., *XC_1311* and *XC_2251*), named Δ*rpoN1rpoN2* (Supplementary Table S1), and the mutants deficient in 9, 10, or 11 σ^70^ family factor-encoding genes, named Δ9, Δ10, or Δ11 (Supplementary Table S1), were constructed by deleting the genes one by one using the same method. The primers used for the construction of these mutants are listed in Supplementary Table S2.

For complementation of the *rpoE1* deletion mutant Δ*rpoE1 in cis*, a 691 bp fragment upstream and a 739 bp fragment downstream of the intergenic region between the ORFs *XC_0742* and *XC_0743* were amplified using the total DNA of *Xcc* strain 8004 as template and the primer sets 0742LF/LR and 0742RF/RR (Supplementary Table S2), respectively. Simultaneously, an 1121 bp fragment containing the *rpoE1* coding region and promoter was amplified with the primer set CC2974F/R (Supplementary Table S2). The 3 fragments were cloned together into the suicide plasmid pK18mobsacB, resulting pK2974CC, which was then introduced into the *rpoE1* deletion mutant Δ*rpoE1* by triparental conjugation and transconjugants were screened on NYG plate supplemented with Rif and 10% sucrose. The obtained cis-complemented mutant strain was confirmed by PCR and named CΔ*rpoE1* (Supplementary Table S1).

### Construction of strains 8004/pJC3806, 8004/pJC3843 and 8004_RpoE1Flag_

To construct strains 8004/pJC3806 and 8004/pJC3843, 2471 and 1476-bp fragments containing the *XC_3806* and *XC_3843* coding regions and promoters were amplified with the primer sets C3806F/R and C3843F/R (Supplementary Table S2), respectively. The obtained fragments were cloned into the plasmid pLAFRJ (Supplementary Table S1) and the resulting plasmids, named pJC3806 and pJC3843, were then introduced into the *Xcc* strain 8004 by triparental conjugation. Transconjugants were selected in NYG medium supplemented with Rif and Tc, confirmed by PCR and named 8004/pJC3806 and 8004/pJC3843 (Supplementary Table S1), respectively.

For ChIP (Chromatin Immunoprecipitation) assay, a strain producing a 3 × Flag-tag fused RpoE1 protein (RpoE1-Flag3) was constructed. A 700 bp fragment upstream and a 682 bp fragment downstream of the ORF *XC_2974* (*rpoE1*) stop codon were amplified using the total DNA of *Xcc* strain 8004 as template and the primer sets Flag2974LF/LR and Flag2974RF/RR (Supplementary Table S2), respectively. The primers were modified to give *Eco*RI- and *Bam*HI- or *Xba*I- and *Hin*dIII-compatible ends. Simultaneously, a DNA fragment encoding 3 × Flag-tag with *Bam*HI- and *Xba*I-compatible ends was synthesized. The three fragments were ligated and cloned into the *Eco*RI and *Hin*dIII sites of the suicide plasmid pK18mobsacB, resulting a recombinant plasmid named pKrpoE1Flag. This plasmid was introduced into *Xcc* strain 8004 by triparental conjugation and transconjugants were screened on selective agar plates containing 10% sucrose. The obtained recombinant strain was further confirmed by PCR and named 8004_RpoE1Flag_ (Supplementary Table S1).

### Construction of promoter reporter plasmid

A promoter-*gusA* transcriptional fusion reporter of *rpoE1* was constructed. A 526 bp DNA fragment from 400 bp upstream to 126 bp downstream of the translational start codon of the RpoE1-encoding ORF *XC_2974* was amplified using the total DNA of *Xcc* strain 8004 as template and the primer set GUS2974F/R (Supplementary Table S2) and fused to the promoterless *gusA* gene with its ribosome binding site in the plasmid pLgus (Supplementary Table S1) in an orientation to allow the *gusA* gene to be driven by the *rpoE1* promoter. The obtained reporter plasmid was confirmed by sequencing and named pLgusrpoE1 (Supplementary Table S1).

### Overproduction and purification of RpoE1-His6 protein

To overproduce 6×His-tagged RpoE1 protein, a 621 bp full length *rpoE1* gene was amplified from the genome of *Xcc* strain 8004 using the primer sets E2974F/R (Supplementary Table S2) and cloned into the expression vector pET30a, generating the recombinant plasmid named pET2974 (Supplementary Table S1), in which *rpoE1* was fused N-terminally and in frame to the 6×His tag-coding region of pET30a. The recombinant plasmid was transformed into *E. coli* strain BL21(DE3), resulting strain BL21(DE3)/pET2974 (Supplementary Table S1). After induction by IPTG, the cells were harvested and the 6×His-tagged RpoE1 protein (RpoE1-His6) was extracted and purified by Nickel-NTA resin.

### Test of enzyme activity and EPS production

GUS activity was determined by measurement of the absorbance of OD_415_ using ρ-nitrophenyl-β-D-glucuronide as the substrate, as described by Jefferson et al. (1986), after growth of *Xcc* strains in minimal medium for 24 h. Three independent experiments were performed and three replicates were used in each experiment. Extracellular amylase, endoglucanase, and protease activity was examined using starch, carboxymethylcellulose, and skimmed milk as substrates, respectively, as described previously (Zang et al., 2007). EPS production was detected by growing bacterial cells on NYG plates containing 2% glucose at 28°*C* for 2 days.

### Analysis of RNA-Seq data

RNA-seq data were analyzed as described by Liu et al. (2013). Differentially expressed genes were determined based on the DESeq package (Anders and Huber, 2010), in which false discovery rate (FDR) was used to determine differentially expressed genes. In this study, genes having FDR ≤ 0.001 and |log_2_FC| (log_2_ of the fold changes) ≥1 (equivalent to two times of fold change) were considered as differentially expressed.

### ChIP assay

*Xcc* cells were grown in 1000 ml of the minimal medium XCM1 for 24 h and cross-linked by adding formaldehyde to a final concentration of 1%. After incubation for 20 min at room temperature with slow shaking, glycine was added at a final concentration of 0.125 M to quench the cross-linking reaction. Bacterial cells were collected by centrifugation at 4°*C* for 5 min at 8,000 g and washed twice in PBS. To lyse the cells, 10 ml of RIPA buffer (50 mM Tris-HCl pH 7.4, 150 mM NaCl, 1 mM EDTA, 1% NP-40, 0.5% sodium deoxycholate) was added and thoroughly mixed by vortexing, and then disrupted by sonication (30 min, 5 s each, with 2 s cooling between each pulse). For each ChIP sample, 50 μl of ANTI-FLAG (agarose conjugated) were added to the bacterial lysates, and incubated with gentle shaking at 4°C overnight. Unbound DNA fragments were washed using RIPA buffer, the bound DNA fragments and proteins were eluted by 0.25 M glycine (pH 2.5).

### Western blotting

Bacterial proteins were separated by SDS-PAGE and were transferred onto a PVDF (polyvinylidene difluoride) membrane. After blocking with 1% milk, the proteins in the membrane were incubated with the 1:1,000 diluted anti-Flag-tag mouse monoclonal antibody as the primary antibody, followed by washing with TBST buffer [Tris 20 mM, NaCl 0.3 M, Tween 20 0.08% (V/V)] for six times. The diluted 1:1,000 horseradish peroxidase (HRP) conjugated goat anti-mouse IgG was used as the secondary antibody. The membrane was washed for six times and luminescent signal was then detected according to the manufacturer's instructions.

### Electrophoresis mobility shift assay (EMSA)

The purified RpoE1-His6 protein was mixed with the 354-bp DNA fragment containing the promoter region of *hrpX*, which was amplified from the genome of *Xcc* strain 8004 by PCR using the FAM-labeled primer set PhrpX-F(FAM)/PhrpX-R(FAM) (Supplementary Table S2), in 20 μl (total volume) of binding buffer (10 mM Tris, 50 mM KCl, 1 mM DTT, pH 7.5). The mixture was incubated for 20 min at room temperature. The samples were then run on a 4% polyacrylamide gel in 0.5 × TBE electrophoresis buffer, and visualized by laser scanning after electrophoresis. A 353-bp DNA fragment containing *hrpG* promoter was also amplified from the genome of strain 8004 using the primer set PhrpG-F(FAM)/PhrpG-R(FAM) (Supplementary Table S2). Whether the DNA fragment interacts with RpoE1-His6 protein was also determined by EMSA in the same way.

### *In vitro* transcription assay

An *in vitro* transcription assay was employed to study the effect of RpoE1 on the expression of *hrpX*. A 647-bp template DNA fragment extending from −438 to +209 relative to the transcriptional initiation site (TIS) of the *hrpX* promoter was amplified by PCR from the genome of *Xcc* strain 8004 using the primers ivt3076F/R (Supplementary Table S2). For the *in vitro* transcription, 2 nM template DNA was incubated with certain amount of RpoE1-His6 protein in the transcription buffer [40 mM Tris-HCl (pH 7.9), 6 mM MgCl_2_, 2 mM spermidine, 10 mM NaCl, 5 mM DTT, 5% glyceol, 50 mM KCl and 1 U RNase inhibitor]. Then, an NTP mixture (250 μM ATP, 250 μM CTP, 250 μM GTP, 20 μM UTP and 250 μM Biotin-16-UTP) and certain amount of *E. coli* holo RNA polymerase or core RNA polymerase were added to start the transcription. After incubation at 37°C for 30 min, the reactions were terminated by the addition of one volume of 2 × RNA Loading Dye Solution and incubation at 70°C for 10 min, and then chilled on ice for 1 min. The transcription products were run on a 4.5% polyacrylamide gel containing 7 M urea in 0.5 × TBE electrophoresis buffer. The transcripts obtained were analyzed by a phosphorimager screen (Typhoon 9410; Amersham Biosciences, Piscataway, NJ, USA). A 582-bp DNA fragment containing *hrpG* promoter, extending from −344 to +238 relative to the *hrpG* TIS, was amplified by PCR from the genome of *Xcc* strain 8004 using the primers ivt3077F/R (Supplementary Table S2) and used in the assay.

### Plant assay

The virulence of *Xcc* strains was tested on the host plant Chinese radish (*Raphanus sativus*) by the leaf-clipping method (An et al., 2017). Briefly, two fully expanded leaves per seedling were cut with scissors dipped in the bacterial suspensions of an OD_600_ of 0.001 (1 × 10^6^ CFU/ml). Thirty leaves were inoculated for each strain in each independent experiment. After being maintained at 100% humidity for 24 h, the inoculated plants were maintained in a greenhouse with 12 h day/night cycle illumination by fluorescent lamps at 25–28°C. Lesion length was measured 10 days after inoculation, and data were analyzed by *t*-test.

To determine the bacterial growth of *Xcc* strains *in planta*, Chinese radish leaves were inoculated by the same method used for the virulence test as described above, and five inoculated leaves for each sampling were homogenized in 9 ml of sterile distilled water. Diluted homogenates were plated on NYG plates supplemented with appropriate antibiotics. Bacterial CFU were counted after incubation at 28°C for 3 days.

The HR was tested on the pepper plant ECW-10R (*Capsicum annuum* cv. ECW-10R), a non-hosts plant commonly used to test the HR of *Xcc*. Bacterial cells from overnight culture were resuspended in sterile distilled water to an OD_600_ of 0.01 (1 × 10^7^ CFU/ml). The pepper leaves were inoculated by infiltrating an ~5-μl bacterial resuspension into the leaf mesophyll tissue by using a blunt-end plastic syringe. The inoculated plants were maintained in a greenhouse with 12 h day/night cycle illumination with fluorescent lamps and a constant temperature of 28°C, and HR symptoms were observed and photographed. At least three plants were inoculated in each experiment, and each experiment was repeated at least three times. For the electrolyte leakage assay, four 0.6 cm^2^ leaf disks for each sample were collected from the bacteria-infiltrated area and incubated in 10 ml of ultrapure water. Conductivity was measured with a DDS-307A conductometer. Three samples were taken for each measurement in each experiment, and each experiment was repeated at least three times.

### Bioinformatic and statistical analysis

The secondary structure of the sigma factors was bioinformatically determined with SMART (Simple Modular Architecture Research Tool) program (http://smart.embl-heidelberg.de). The homologs of sigma factors were obtained from the KEGG database (https://www.kegg.jp/) and by a BLAST against the NCBI database (https://blast.ncbi.nlm.nih.gov/). Student's *t*-test was used to determine the statistical significance of differences between the means of at least three technical replicates in an experiment. A *P*-value ≤ 0.05 was considered statistically significant.

## Results

### Characterization of σ factor signaling systems in *Xcc*

Fifteen putative σ factors were identified within the proteome of *Xcc* strain 8004 using the simplified modular architecture research tool (SMART) program and blast analysis (http://smart.embl-heidelberg.de) (Table 1). SMART analysis revealed two proteins (XC_1311 and XC_2251) as members of σ^54^ family and the rest are σ^70^ family (Table 1). Of the σ^70^ family members, one (XC_3806), two (XC_3843 and XC_2281), and 10 (XC_0556, XC_1193, XC_1474, XC_2566, XC_2905, XC_2934, XC_2974, XC_3099, XC_3383 and XC_3864) belong to group I, group III, and group IV, respectively (Table 1). Notably, only 14 σ factors in the genome of the strain were annotated and XC_3864 was predicted as a hypothetical protein (Qian et al., 2005, Table 2). The σ^70^ group I member XC_3806 is highly homologous to the housekeeping σ factor RpoD, while the group III members XC_3843 and XC_2281 are highly homologous to the housekeeping σ factor RpoH and the flagellar σ factor FliA of *E. coli* (Table 2). Apart from XC_2974 which was annotated as RpoE, all other group IV members were only annotated as sigma factors (Qian et al., 2005, Table 2). Given that all the group IV members have a motif similar to RpoE, we named XC_2974 RpoE1 and the others RpoE2 to RpoE10 based on the annotation of *Xcc* strain B100 and *X. campestris* pv. *vesicatoria* strain 85-10 (Table 2). Importantly, homologs of these predicted σ factors were found in other bacterial plant pathogens. All were highly conserved among sequenced *Xcc* strains (Table 2). The homologs of the two predicted σ^54^ factors XC_1311 (RpoN1) and XC_2251 (RpoN2) are found in all species of *Xanthomonas* and *Ralstonia solanacearum*, but only XC_1311 homolog is found in other plant associated bacterial species of *Pseudomonas* and *Erwinia* (Table 2).

**Table 1:** The ORFs annotated as sigma factors in *Xcc* strain 8004^*^.

	**ID (gene name)**	**Motif analysis**
σ^70^ **FAMILY**		
Group 1	*XC_3806 (rpoD)*	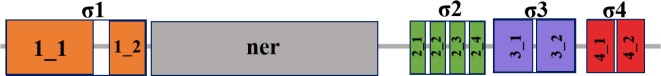
Group 3	*XC_3843 (rpoH)*	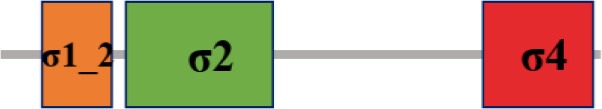
	*XC_2281 (fliA)*	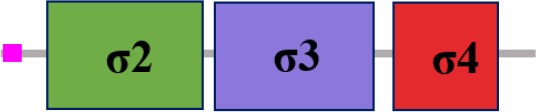
Group 4	*XC_2974 (rpoE1)*	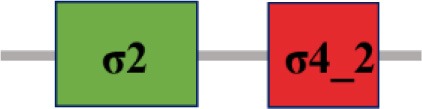
	*XC_3383 (rpoE2)*	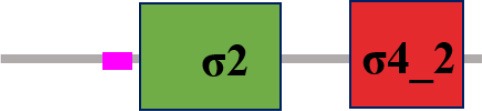
	*XC_2905 (rpoE3)*	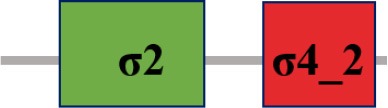
	*XC_2566 (rpoE4)*	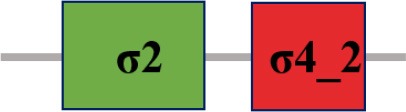
	*XC_1474 (rpoE5)*	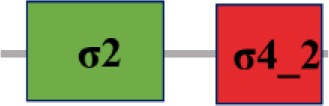
	*XC_1193 (rpoE6)*	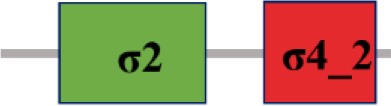
	*XC_2934 (rpoE7)*	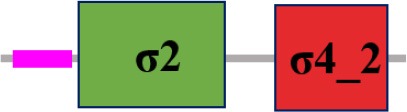
	*XC_3864 (rpoE8)*	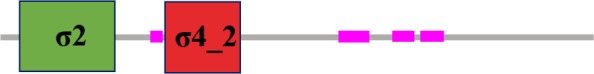
	*XC_0556 (rpoE9)*	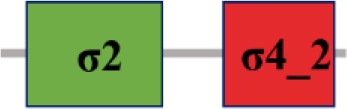
	*XC_3099 (rpoE10)*	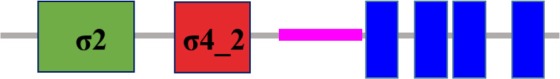
σ^54^ Family	*XC_1311 (rpoN1)*	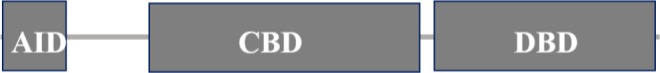
	*XC_2251 (rpoN2)*	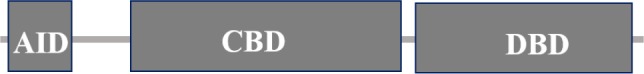

* *The protein sequence of σ^70^ family members can be divided into four regions (σ_1_, σ_2_, σ_3_, σ_4_) which are further divided into subregions on the basis of sequence conservation. A σ^54^ protein is divided into three regions (AID, CBD, DBD). Ner, non-essential region; 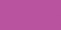, Low complexity region; 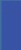: Transmembrane domain*.

**Table 2:** Distribution of the 15 putative σ factors among other phytopathogenic bacteria^*^.

	**Rename in *Xcc* strain 8004**	***Xanthomonas***							***Pseudomonas***		***Ralstonia***	***Erwinia***
		**Annotation in** Xcc^**^			***Xcv***	***Xac***	***Xoo***	***Xoc***	***Pst***	***Pa***	***Rs***	***Ea***
		**8004**	**ATCC 33913**	**B100**	**85–10**	**306**	**PXO99**	**BLS256**	**DC3000**	**PAO1**	**GMI1000**	**CFBP1430**
σ^70^ **FAMILY**												
Group 1	*XC_3806 rpoD*	*XC_3806 rpoD*	*XCC3736* (100) *rpoD*	*xcc-b100_3917* (98.4) *rpoD*	*XCV3912* (97.6) *rpoD*	*XAC3788* (89.2) *rpoD*	*PXO_04069* (97.1) *rpoD*	*XOC_4120* (97.6) *rpoD*	*PSPTO_0537* (62.2) *rpoD*	*PA0576* (62.9) *rpoD*	*RSc2215* (56.2) *rpoD*	*EAMY_0415* (60.6) *rpoD*
Group 3	*XC_3843 rpoH*	*XC_3843 rpoH*	*XCC3771* (100) *rpoH*	*xcc-b100_3953* (100) *rpoH*	*XCV3949* (98.3) *rpoH*	*XAC3824* (98.3) *rpoH*	*PXO_03711* (98.9) *rpoH*	*XOC_0605* (98.6) *rpoH*	*PSPTO_0430* (58.7) *rpoH*	*PA0376* (58.0) *rpoH*	*RSc0374* (56.1) *rpoH*	*EAMY_3492* (58.8) *rpoH*
	*XC_2281 fliA*	*XC_2281 fliA*	*XCC1906* (100) *fliA*	*xcc-b100_2201* (100) *fliA*	*XCV1977* (98.8) *fliA*	*XAC1933* (99.2) *fliA*	*PXO_00957* (97.2) *fliA*	*XOC_2329* (98.8) *fliA*	*PSPTO_1979* (54.3) *fliA*	*PA1455* (50.8) *fliA*	*RSp1390* (47.2) *fliA*	*EAMY_2139* (49.6) *fliA*
Group 4	*XC_2974 rpoE1*	*XC_2974 rpoE*	*XCC1267* (100) *rpoE*	*xcc-b100_3036* (100) *rpoE*	*XCV1370* (97.6) *rpoE2*	*XAC1319* (97.6) *algU*	*PXO_01711* (95.6) *rpoE*	*XOC_3188* (97.6)	*PSPTO_4224* (61.7) *rpoE*	*PA0762* (62.6) *algU*	*RSc1055* (58.4) *rpoE*	*EAMY_2631* (55.8) *rpoE*
	*XC_3383 rpoE2*	*XC_3383* σ^70^	*XCC0847* (100) σ^70^	*xcc-b100_3503* (100) σ^70^	*XCV0954* (86.9) *rpoE1*	*XAC0922* (87.3)	*PXO_04572* (88.4)	*XOC_0976* (88.5)	N	N	*RSc2078* (40.2)	N
	*XC_2905 rpoE3*	*XC_2905* σ^70^	*XCC1334* (100) *rpoE*	*xcc-b100_2963* (100) σ^70^	*XCV1436* (95.2) *rpoE3*	*XAC1380* (95.2) *rpoE*	*PXO_01782* (95.7)	*XOC_3114* (94.1)	*PSPTO_5176* (34.8)	N	*RSc2361* (40.0)	*EAMY_0593* (28.0)
	*XC_2566 rpoE4*	*XC_2566* σ^70^	*XCC1665* (99.0) *rpoE*	*xcc-b100_2594* (99.5) *rpoE4*	*XCV1718* (93.3) *rpoE4*	*XAC1682* (93.3) *rpoE*	*PXO_00212* (93.3)	*XOC_2834* (93.8)	N	N	N	N
	*XC_1474 rpoE5*	*XC_1474* σ^70^	*XCC2643* (100) *rfaY*	*xcc-b100_2995* (26.9) *rpoE5*	*XCV2975* (75.2) *rpoE5*	*XAC2814* (77.8) *rfaY*	*PXO_04771* (69.9)	*XOC_1631* (77.2)	N	N	N	N
	*XC_1193 rpoE6*	*XC_1193* σ^70^	*XCC2916* (100) σ^70^	*xcc-b100_1237* (97.9) σ^70^	*XCV3224* (89.7) *rpoE6*	N	N	N	N	*PA2896* (38.2)	N	N
	*XC_2934 rpoE7*	*XC_2934* σ^70^	*XCC1306* (100) *algU*	*xcc-b100_1518* (19) σ^70^	N	N	N	*XOC_3146* (77.2)	N	N	N	N
	*XC_3864 rpoE8*	*XC_3864* hypothetical protein	*XCC3792* (97.6) hypothetical protein	*xcc-b100_3976* (97.9) σ^70^	N	N	N	N	N	*PA1351* (45.6)	*RSp0636* (61.8)	N
	*XC_0556 rpoE9*	*XC_0556* σ^70^	*XCC3593* (100) *fecI*	*xcc-b100_0572* (100) σ^70^	*XCV4222* (31.2) *rpoE9*	*XAC4129* (30.8) *rpoE*	N	N	*PSPTO_1209* (47.9)	*PA3899* (52.4)	*RSp0849* (42.4) *prhI*	N
	*XC_3099 rpoE10*	*XC_3099* σ^70^	*XCC1143* (100) *rfaY*	*xcc-b100_3196* (99.8) σ^70^	*XCV1276* (70.8) *rfaY*	N	*PXO_04856* (76.8)	*XOC_3475* (76.1)	N	N	N	N
σ^54^ Family	*XC_1311 rpoN1*	*XC_1311* σ^54^	*XCC2802* (100) *rpoN*	*xcc-b100_1358* (100) *rpoN1*	*XCV3118* (93.1) *rpoN2*	*XAC2972* (93.5) *rpoN2*	*PXO_02227* (93.8) *rpoN1*	*XOC_1439* (93.7) *rpoN*	*PSPTO_4453* (42.5) *rpoN*	*PA4462* (43.2) *rpoN*	*RSc0408* (42.4) *rpoN1*	*EAMY_0316* (42.4) *rpoN*
	*XC_2251 rpoN2*	*XC_2251* σ^54^	*XCC1935* (100) *rpoN*	*xcc-b100_2232* (100) *rpoN2*	*XCV2016* (91.6) *rpoN1*	*XAC1969* (92.3) *rpoN1*	*PXO_00995* (93.1) *rpoN2*	*XOC_2368* (92.9) *rpoN*	N	N	Rsp1671 (37.8) *rpoN2*	N

* *Numbers in parentheses represent percentages of amino-acid identity; N denotes no homologous (or similar) protein was found*.

** *Xcc, X. campestris pv. campestris; Xcv, X. campestris pv. vesicatoria; Xac, X. axonopodis pv. citri; Xoo, X. oryzae pv. oryzae; Xoc, X. oryzae pv. oryzicola; Pst, P. syringae pv. tomato; Pa, P. aeruginosa; Rs, R. solanacearum; Ea, E. amylovora*.

### RpoE1 is a putative σ^70^ factor which influences *Xcc* pathogenesis

To investigate whether these putative σ factors are involved in the regulation of pathogenesis we constructed a panel of strains defective for each gene encoding an individual σ factor. These deletion mutants were generated by a double-crossover homologous recombination strategy using the suicide plasmid pK18*mobsacB*. Only 13 of the 15 predicted σ factor mutants were obtained in *Xcc* 8004. Despite several attempts strains with *XC_3806* or *XC_3843* successfully deleted could not be attained. Given that no mutant strain could be generated for *XC_3806* or *XC_3843* and that the proteins encoded by these two ORFs are highly homologous to the housekeeping σ factors RpoD and RpoH, it is very possible that these genes might play a role in the viability of *Xcc*. To verify this, we performed the deletion mutagenesis in the recombinant strains 8004/pJC3806 and 8004/pJC3843 respectively. These strains were constructed by introducing the vector pLAFRJ containing a copy of *XC_3806* or *XC_3843* respectively into the wild-type strain 8004 (Supplementary Table S1). This allows the chromosomal copy of *XC_3843* or *XC_3806* to be deleted (Supplementary Figure S1). This result provides evidence demonstrating that *XC_3843* (*rpoD*) or *XC_3806* (*rpoH*) are housekeeping genes in *Xcc* strain 8004.

**Figure 1:**
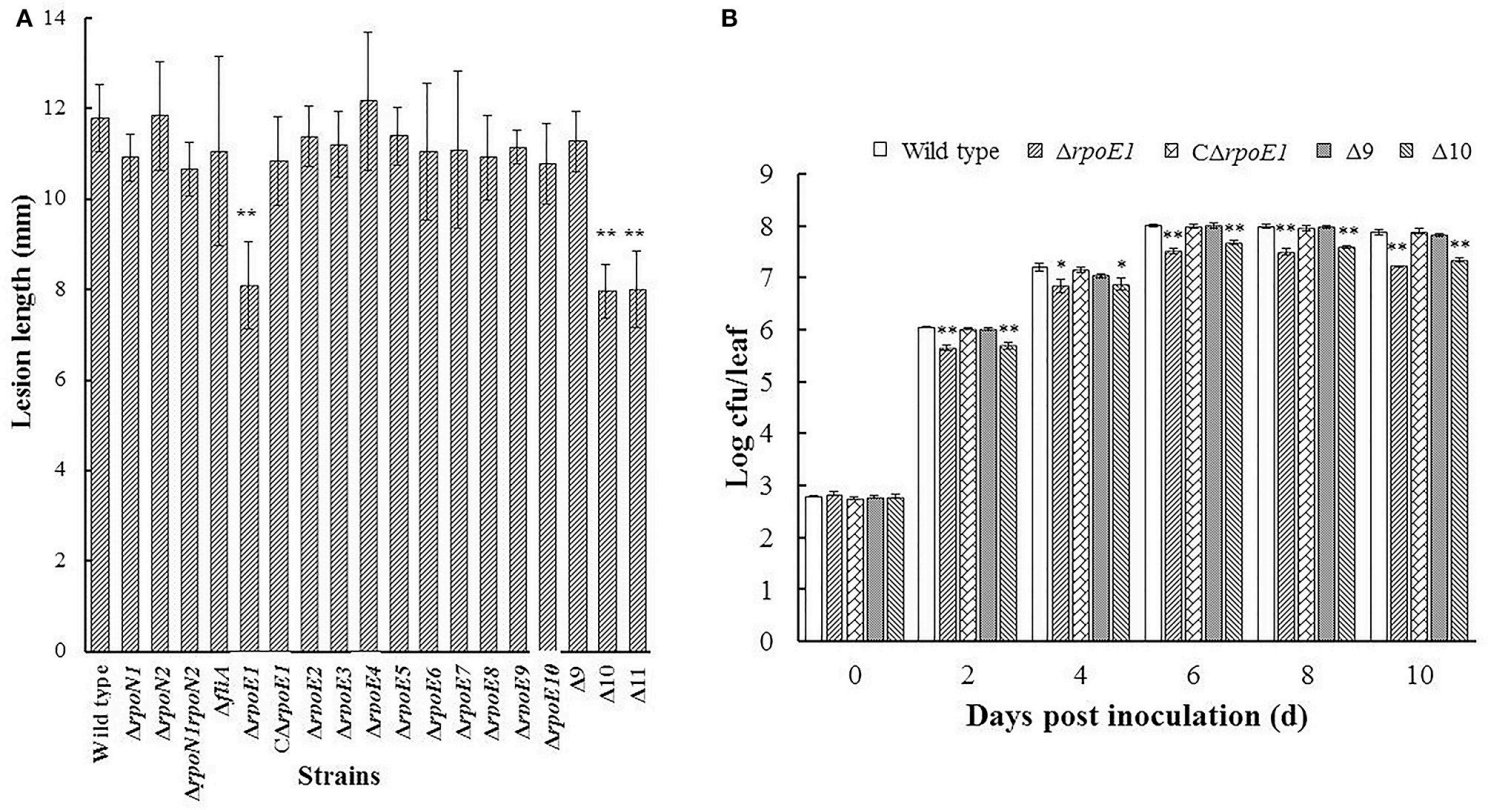
RpoE1 is required for full virulence of *Xcc*. Bacterial cells of *Xcc* strains from overnight culture were washed and re-suspended in sterile distilled water to an OD_600_ of 0.001 (1 × 10^6^ CFU/ml). Inoculation was carried out by cutting the leaves of the host plant Chinese radish with scissors dipped in the bacterial re-suspensions. **(A)** Mean lesion lengths caused by the *Xcc* strains tested. The lesion lengths were measured at 10 days post-inoculation. The data represent the means and standard deviations from 30 inoculated leaves. **(B)** Bacterial populations of *Xcc* strains in the inoculated leaves. Five inoculated leaves for each strain were taken and homogenized in sterile water. The homogenates were diluted and plated on NYG plates. Bacterial CFUs were counted after incubation for 3 days. Data are the means and standard deviations from three replicates. The experiment was repeated three times with similar results. Asterisks indicate statistically significant difference, compared with the wild type (Student's *t*-test. ^*^P < 0.05; ^**^P < 0.01).

In parallel, mutants were tested for their impact on virulence *in planta* using the Chinese radish leaf-clipping assay. Ten days post-inoculation the majority of mutants tested produced disease symptoms similar to the wild-type strain 8004 with mean lesion lengths from 10.78 to 12.17 mm (*t*-test, *P* > 0.1) (Figure 1A, Supplementary Table S3). However, in the case of strain Δ*rpoE1* (*XC_2974*) it showed reduced disease symptoms compared to wild-type with a mean lesion length of 8.09 mm which was significant (*t*-test, *P* = 0.007) (Figure 1A, Supplementary Table S3). Furthermore, examining the growth rate of strain Δ*rpoE1* in the host plant revealed that bacterial cell number (cfu, colony forming units) was lower than that of wild type (Figure 1B). Importantly, phenotypes of the mutant strain Δ*rpoE1* for virulence and *in planta* growth could be restored toward the wild-type phenotype by introduction vector expressing *rpoE1* (CΔ*rpoE1*) (Figure 1). These data suggest that the σ^70^ factor RpoE1 is required for *Xcc* full virulence. The growth rate of Δ*rpoE1* in the nutrient rich medium NYG or the minimal medium XCM1 was similar to the wild type (Supplementary Figure S2), suggesting that mutation of *rpoE1* did not have an impact on *Xcc* growth.

Although RpoE1 had a strong influence on virulence the other individual σ^70^ factors did not appear to contribute to virulence regulation (Figure 1). However, it could not be excluded that several of these σ^70^ factors may have redundant functions and/or collectively contribute to virulence. As a result individual mutations would not reveal any insight of their regulation. To evaluate whether any of other σ^70^ factor-encoding genes may have a collective effect on *Xcc* virulence, we constructed a mutant strains, named Δ9, Δ10, and Δ11 (Supplementary Table S1). The Δ9 strain has the other nine σ^70^ factor-encoding genes (except *rpoE1, rpoD* and *rpoH*) deleted sequentially, while the Δ10 also had *rpoE1* deleted and Δ11 the additional σ^70^ factor-encoding gene deleted. The Δ9 and Δ10 strains were tested for virulence using the Chinese radish leaf-clipping assay (Figure 1). The Δ9 showed similar disease symptoms and *in planta* growth rate to the wild-type strain (Figure 1). However, the mutant Δ10 and Δ11 displayed similar disease symptoms and *in planta* growth rate to the mutant strain Δ*rpoE1* but no further additive effect was seen (Figure 1). The double deletion mutant strain (named Δ*rpoN1rpoN2*) of the σ^54^ family members RpoN1 and RpoN2 could still induce wild-type disease symptoms (Figure 1A). Taken together, it is clear that RpoE1, RpoD, and RpoH play a significant role in *Xcc* physiology and pathogenesis. However, no roles in virulence for the other σ^54^ or σ^70^ factors were revealed under the tested conditions.

### RpoE1 influences T3SS but not extracellular enzymes or extracellular polysaccharides secretion

*Xcc* is a very adaptable pathogen and employs several virulence mechanisms and factors when causing disease. These include secreted factors like extracellular enzymes (such as protease, endoglucanase, and amylase) and the extracellular polysaccharides (EPS) that contribute collectively to *Xcc* virulence (Ryan et al., 2011, 2015; Vicente and Holub, 2013; Zhou et al., 2017). The most study of virulence mechanisms in *Xcc* is the T3SS that translocates effector proteins into host cells and is essential for disease. *Xcc* effector proteins induce disease symptoms on susceptible host plants and the hypersensitive response (HR) on resistant host or non-host plants (Ryan et al., 2011, 2015; Vicente and Holub, 2013; Zhou et al., 2017). To gain an insight into the specific virulence functions that the σ^70^ factor RpoE1 may influence in *Xcc* we examined EPS production, extracellular enzyme activity as well as HR induction. To do this we compared mutant strain Δ*rpoE1* with those of the wild-type. The results demonstrated that Δ*rpoE1* and the wild-type produced comparable levels of EPS, extracellular protease, endoglucanase, and amylase activities (Supplementary Figure S3), suggesting that RpoE1 is not involved in the production of these virulence factors.

**Figure 2:**
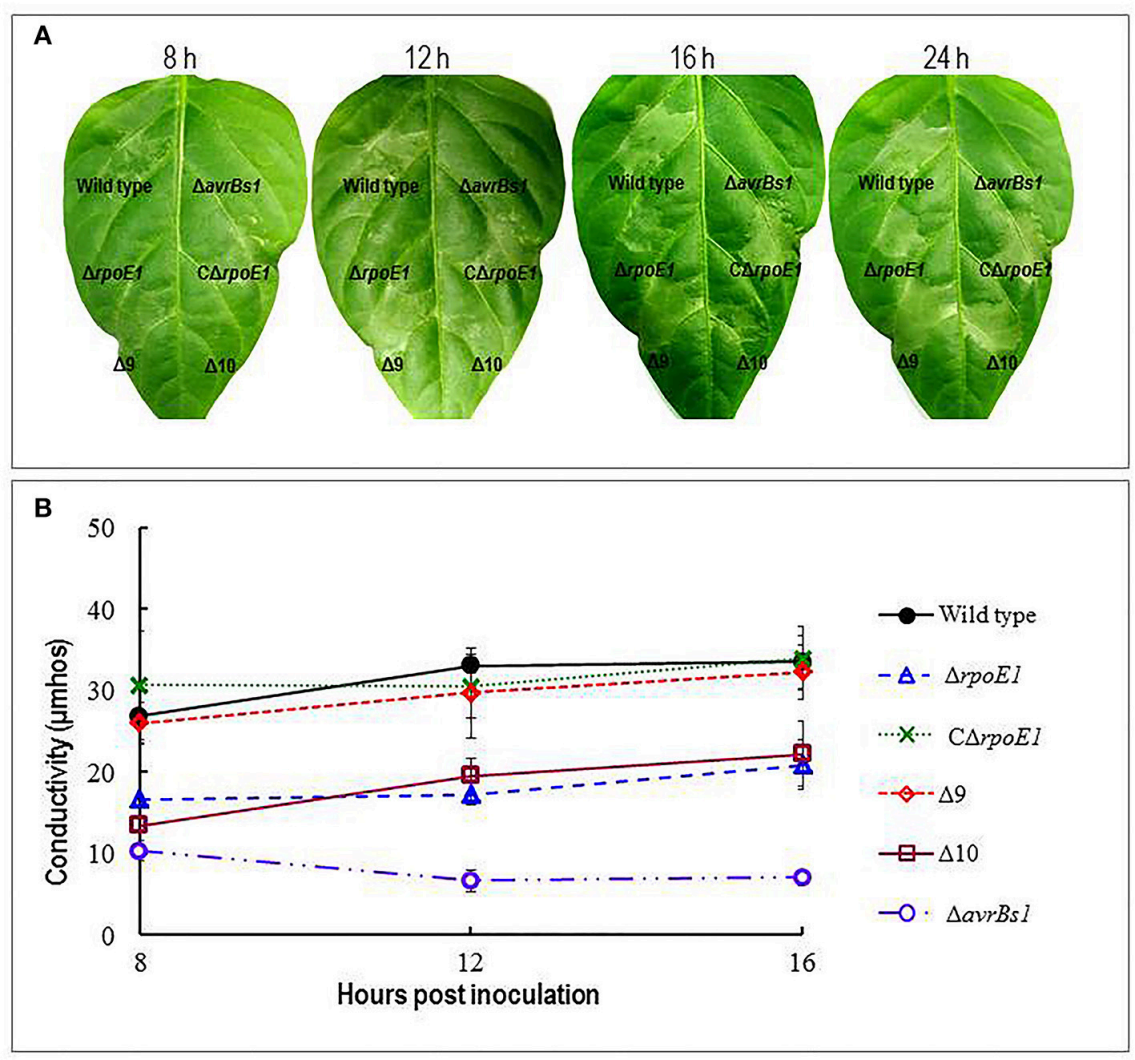
RpoE1 contributes to hypersensitive response (HR) of *Xcc*. **(A)** Bacterial cells of *Xcc* strains from overnight culture were washed and re-suspended in sterile distilled water to an OD_600_ of 0.01 (1 × 10^7^ CFU/ml). Approximately 5 μl bacterial re-suspension was infiltrated into the leaf mesophyll tissue of the non-host plant pepper ECW-10R with a blunt-end plastic syringe. Pictures of the inoculated pepper leaves were taken at 8, 12, 16, and 24 h after infiltration. **(B)** Electrolyte leakage from the pepper leaves inoculated was determined. Four 0.6 cm^2^ leaf disks for each sample were collected from the infiltrated area and incubated in 10 ml of ultrapure water. Conductivity was measured with a DDS-307A conductometer. Three samples were taken for each measurement in each experiment, and each experiment was repeated at least three times. The results presented are from a representative experiment, and similar results were obtained in all other independent experiments.

To examine if RpoE1 is involved in the T3SS, the mutant strain Δ*rpoE1* was examined for its ability to induce HR in the pepper cultivar ECW-10R (*Capsicum annuum* cv. ECW-10R). The wild-type strain 8004 carries the T3SS effector AvrBs1 and elicits visible HR symptoms on the leaves 8 h after inoculation (Xu et al., 2008). In this study, after 8 h post inoculation the Δ*rpoE1* strain triggered a delayed and weakened HR, compared to the wild type (Figure 2A). Furthermore, the Δ*avrBs1* strain, an AvrBs1-defective mutant, was also unable to elicit visible HR symptoms in the pepper cultivar ECW-10R (Supplementary Table S1). Interestingly, Δ*rpoE1* strain could elicit visible HR symptoms 16 h post-inoculation. However, the HR symptoms were weaker compared to those elicited by the wild-type (Figure 2A). Importantly, the complemented strain CΔ*rpoE1* caused similar HR symptoms to the wild-type 8 h after inoculation (Figure 2A), indicating that the HR-induction capability of Δ*rpoE1* could be restored by *rpoE1 in cis* complementation.

These observations were substantiated using an electrolyte leakage assay. Where the Δ*rpoE1* mutant displayed significantly lower electrolyte leakages at 8, 12, and 16 h after inoculation compared with the wild-type (Figure 2B). Similar to the leaf clipping virulence assays the mutant Δ9 and Δ10 were tested along with the Δ*rpoE1* strain. Like the leaf clipping assays the Δ10 demonstrated similar impact on HR and electrolyte leakage as the Δ*rpoE1* strain, while the Δ9 mirrored the wild-type strain phenotypes (Figure 2). Taken together, these data suggest that the σ^70^ factor RpoE1 is involved in the T3SS but not extracellular enzymes or EPS secretion.

### Global transcriptome profiling reveals the extended scope of regulation by σ^70^ factor RpoE1 in *Xcc*

One of the important features of bacterial σ^70^ factor is the activation of selective gene transcription depending on the environmental conditions (Kazmierczak et al., 2005; Davis et al., 2017). To gain an understanding of the impact of RpoE1 on *Xcc* gene transcription we compared the global transcriptional profile of *rpoE1* deletion mutant (Δ*rpoE1* strain) with the wild-type. These strains were grown in minimal medium XCM1 as it mimics to an extent the conditions *in planta*, given that genes involved in virulence such as the *hrp* cluster (T3SS) are active (Jiang et al., 2013). The total RNA was isolated from planktonic cultures growing in exponential phase. Three biological replicates were analyzed. This transcriptome profile analysis revealed that of the 4273 annotated genes, 131 genes were found to be differentially expressed; of which, 21 and 110 were up- and down-regulated, respectively (Table 3). To confirm the expression changes, semi-quantitative RT-PCR (sqRT-PCR) was employed to analyze selected genes. For the 10 selected genes expression was consistent with changes seen in the global transcriptome analysis (Table 3).

**Table 3:** Genes whose expression was altered in the *rpoE1* deletion mutant ΔrpoE1^*^.

**Gene ID**	**log2 Ratio (Δ*rpoE1*/WT)**	**Predicted product**	**sqRT-PCR results WT Δ*rpoE1***
*XC_0117*	+1.044	Hypothetical protein	
*XC_0118*	+1.007	Transcriptional regulator	
*XC_1263*	+1.155	MFS transporter	
*XC_1298*	+1.904	Pectate lyase II	
*XC_1386*	+1.114	Oxidoreductase	
*XC_1514*	+1.01	Extracellular protease	
*XC_1515*	+1.309	Extracellular protease	
*XC_1789*	+1.251	Glutathione S-transferase	
*XC_2580*	+1.462	Endonuclease	
*XC_2772*	+1.617	Serine peptidase	
*XC_2782*	+1.002	Hypothetical protein	
*XC_2838*	+1.487	Multidrug resistance efflux pump	
*XC_2839*	+1.409	Outer membrane efflux protein	
*XC_3054*	+1.797	endo-1,3-beta-glucanase	
*XC_3549*	+2.831	Hypothetical protein	
*XC_3550*	+2.599	Serine protease	
*XC_3575*	+1.550	Protease	
*XC_3576*	+1.599	Outer membrane protein	
*XC_3591*	+3.184	Pectate lyase	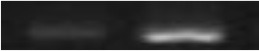
*XC_3956*	+2.324	Hypothetical protein	
*XC_4318*	+2.021	Avirulence protein AvrXccA1	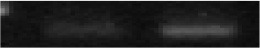
*XC_0052*	−1.233	Avirulence protein AvrBs2	
*XC_0241*	−1.735	Type III effector XopXccN	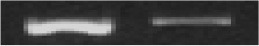
*XC_0253*	−1.088	Dipeptidyl anminopeptidase	
*XC_0268*	−1.422	Putative type III effector protein XopR	
*XC_0334*	−1.297	Transcriptional regulator, MarR family	
*XC_0361*	−1.605	MFS transporter	
*XC_0419*	−1.365	Hypothetical protein	
*XC_0431*	−1.202	VirK protein	
*XC_0519*	−1.671	Ice nucleation protein	
*XC_0541*	−1.101	Conserved hypothetical protein	
*XC_0542*	−1.502	Conserved hypothetical protein	
*XC_0563*	−1.409	Conserved hypothetical protein	
*XC_0705*	−1.297	Endopolygalacturonase	
*XC_0784*	−1.002	Cellulase S	
*XC_0817*	−1.741	Hypothetical protein	
*XC_0932*	−1.082	Hypothetical protein	
*XC_0933*	−1.170	Hypothetical protein	
*XC_1084*	−5.523	Hypothetical protein	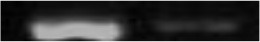
*XC_1111*	−1.801	Cytochrome P450 hydroxylase	
*XC_1146*	−1.754	Hypothetical protein	
*XC_1147*	−2.794	Hypothetical protein	
*XC_1210*	−1.268	Conserved hypothetical protein	
*XC_1422*	−1.336	Cysteine protease	
*XC_1441*	−1.594	Hypothetical protein	
*XC_1442*	−2.238	Serine protease	
*XC_1445*	−2.271	Oxidoreductase	
*XC_1446*	−1.581	Oxidoreductase	
*XC_1447*	−2.795	Serine protease	
*XC_1448*	−2.502	Hypothetical protein	
*XC_1449*	−2.639	Serine protease	
*XC_1450*	−2.678	Serine protease	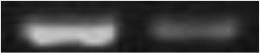
*XC_1553*	−1.756	Avirulence protein AvrAC	
*XC_1713*	−1.391	Hypothetical protein	
*XC_1715*	−1.176	Peptidase	
*XC_1740*	−1.728	Hypothetical protein	
*XC_1811*	−1.762	Virulence protein	
*XC_1849*	−2.003	Polygalacturonase	
*XC_2004*	−1.847	Avirulence protein AvrXccC	
*XC_2081*	−1.251	Avirulence protein AvrBs1	
*XC_2082*	−1.671	Tyrosine phosphatase	
*XC_2405*	−1.341	Transport transmembrane protein	
*XC_2406*	−1.785	Hypothetical protein	
*XC_2407*	−1.822	Hypothetical protein	
*XC_2408*	−2.099	Hydroxyproline-rich glycoprotein DZ-HRGP	
*XC_2409*	−1.989	Hypothetical protein	
*XC_2410*	−1.982	Hypothetical protein	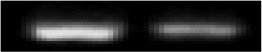
*XC_2411*	−1.372	Hypothetical protein	
*XC_2412*	−1.383	Hypothetical protein	
*XC_2413*	−1.377	Type IV secretion system NTPase VagA	
*XC_2414*	−2.028	Hypothetical protein	
*XC_2415*	−2.229	Hypothetical protein	
*XC_2416*	−2.427	Hypothetical protein	
*XC_2417*	−1.289	Plasmid mobilization protein	
*XC_2512*	−1.794	TonB-dependent receptor	
*XC_2546*	−1.564	MFS transporter	
*XC_2547*	−1.671	ABC transporter ATP-binding protein	
*XC_2602*	−1.551	Avirulence protein AvrXccE1	
*XC_2691*	−1.554	Hypothetical protein	
*XC_2827*	−1.326	MarR family transcriptional regulator	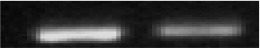
*XC_2972*	−2.373	Periplasmic protease	
*XC_2973*	−2.325	Regulatory protein	
*XC_2974*	−18.599	RNA polymerase sigma factor RpoE2	
*XC_2995*	−1.925	Type III effector XopXccE1	
*XC_2996*	−1.738	Hypothetical protein	
*XC_2998*	−1.785	Hypothetical protein	
*XC_2999*	−2.385	Hypothetical protein	
*XC_3000*	−3.919	Hypothetical protein	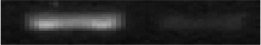
*XC_3001*	−3.048	Hpa2 protein	
*XC_3002*	−2.958	Hpa1 protein	
*XC_3003*	−2.535	HrcC protein	
*XC_3004*	−2.437	HrcT protein	
*XC_3005*	−2.128	HrpB7 protein	
*XC_3006*	−2.536	HrcN protein	
*XC_3007*	−2.444	HrpB5 protein	
*XC_3008*	−2.407	HrpB4 protein	
*XC_3009*	−2.384	HrcJ protein	
*XC_3010*	−2.418	HrpB2 protein	
*XC_3011*	−2.552	HrpB1 protein	
*XC_3012*	−2.205	HrcU protein	
*XC_3013*	−2.232	HrcV protein	
*XC_3014*	−2.401	HpaP protein	
*XC_3015*	−2.164	HrcQ protein	
*XC_3016*	−2.533	HrcR protein	
*XC_3017*	−2.543	HrcS protein	
*XC_3018*	−2.428	HpaA protein	
*XC_3019*	−2.466	HrpD5 protein	
*XC_3020*	−2.205	HrpD6 protein	
*XC_3021*	−2.398	HrpE protein	
*XC_3022*	−2.481	HpaB protein	
*XC_3023*	−2.196	HrpW protein	
*XC_3024*	−2.441	Hypothetical protein	
*XC_3025*	−2.583	HrpF protein	
*XC_3076*	−1.226	HrpX protein	
*XC_3147*	−1.778	Hypothetical protein	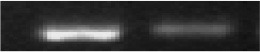
*XC_3148*	−1.557	DNA polymerase III subunit alpha	
*XC_3159*	−1.232	Beta-glucosidase	
*XC_3160*	−1.858	Type III effector XopXccR	
*XC_3176*	−1.369	Type III effector protein	
*XC_3177*	−1.810	Type III effector XopXccQ	
*XC_3178*	−1.009	Hypothetical protein	
*XC_3425*	−1.177	Transcriptional regulator	
*XC_3426*	−1.329	Protocatechuate 4,5-dioxygenase subunit alpha	
*XC_3427*	−1.331	Protocatechuate 4,5-dioxygenase subunit beta	
*XC_3676*	−1.268	Chorismate mutase	
*XC_3802*	−1.402	Avirulence protein AvrXccB	
*XC_3895*	−1.458	Disulfide-isomerase	
*XC_3922*	−2.161	Hypothetical protein	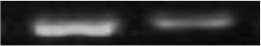
*XC_4206*	−1.495	Hypothetical protein	
*XC_4273*	−1.615	Type III effector XopXccLR	
*XC_4326*	−1.226	Phosphatase	
16S rRNA			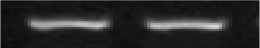

* *WT, wild type strain; “+” and “–” represent genes whose expression was increased and decreased in the rpoE1 deletion mutant ΔrpoE1, compared to the wild type strain 8004*.

Of the 131 differentially expressed genes, 39 were predicted to encode hypothetical proteins (Table 3) with their functions still remaining to be investigated in *Xcc*. Interestingly, 51 of the down-regulated genes are grouped in three large clusters, i.e., *XC_1441-XC_1450* (except *XC_1443* and *XC_1444*) (9.022 kb, lies between nucleotides 1738592 and 1747613 in the genome), *XC_2405-XC_2417* (16.425 kb, lies between nucleotides 2912173 and 2928598 in the genome), and *XC_2995-XC_3025* (except *XC_2997*) (27.626 kb, lies between nucleotides 3591815 and 3619441 in the genome) (Table 3). Cluster A (*XC_1441-XC_1450*) comprises of four-serine protease, one oxidoreductase and two hypothetical protein genes and one pseudogene (Table 3). It is known that the major extracellular protease PrtA, encoded by *XC_3379*, responsible for almost all extracellular protease activity of *Xcc* strain 8004. Inactivation of *prtA* leads to almost complete loss of extracellular protease activity (Meng et al., 2011). Therefore, it is not surprising that deletion of *rpoE1* did not reduce significantly the extracellular protease activity, although RpoE1 regulates four serine protease genes but not *prtA*. Of the 13 genes in cluster B (*XC_2405-XC_2417*), nine encode hypothetical proteins and the others encode transport transmembrane protein, hydroxyproline-rich glycoprotein, putative NTPase, and plasmid mobilization protein, respectively (Table 3). Surprisingly, cluster B is absent in other *Xcc* strains and is probably a recent horizontally acquired DNA segment in strain 8004, as the segment is linked with mobile genetic elements (ORFs *XC_2402* and *XC_2417*) (da Silva et al., 2002; Qian et al., 2005; He et al., 2007; Vorhölter et al., 2008). Importantly for virulence, cluster C (*XC_2995-XC_3025*) includes the *hrp* genes which includes 24 well-defined *hrp, hrc* (*hrp-*conserved), and *hpa* (*hrp-*associated) genes as well as one type III effector gene (*XC_2995*) (Table 3). Furthermore, the down-regulated effector genes include the *avrBs1* (*XC_2081*) which is shown to contribute to HR in pepper cultivar ECW-10R (Xu et al., 2008). Additionally, *XC_3076*, which encodes the *hrp* master regulator HrpX is also down-regulated (Table 3).

Taken together, data reveals that deletion of *rpoE1* significantly decreased the expression of *hrp/hrc/hpa* and type III effector genes, suggesting that RpoE1 plays a positive role in the regulation during conditions that mimic *in planta* infection.

### RpoE1 influences the expression of *hrp/hrc/hpa* and Type III effector genes via the master regulator HrpX

**Figure 3:**
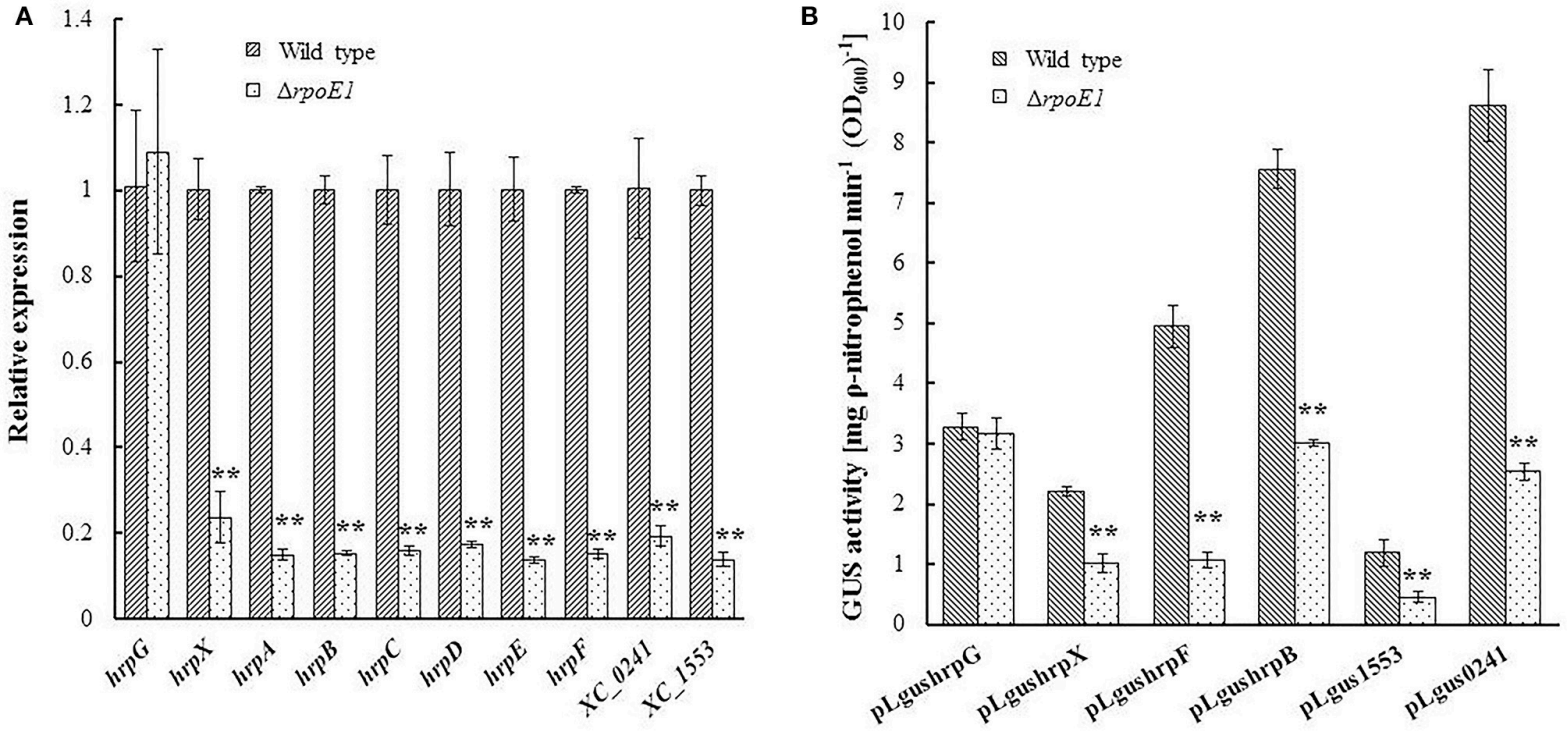
RpoE1 regulates positively the expression of *hrp* gene cluster (*hrpA*-*F*), type III secreted effector-encoding genes (*XC_0241* and *XC_1553*) and the *hrp* master regulator *hrpX*. **(A)** Quantitative real-time PCR analysis of the transcription of *hrpA*-*F, hrpG, hrpX, XC_0241*, and *XC_1553* in the *Xcc* wild-type strain 8004 and the *rpoE1* deletion mutant strain Δ*rpoE1*. RNA was isolated from cultures of the strains grown in XCM1 medium for 24 h. The relative mRNA level was calculated with respect to the level of the corresponding transcript in the wild-type strain 8004. **(B)** GUS activity of *hrpB, hrpF, hrpG, hrpX, XC_0241*, and *XC_1553* promoter-*gusA* reporters (pLgushrpB, pLgushrpF, pLgushrpG, pLgushrpX, pLgus0241, and pLgus1553) in the wild-type strain 8004 and the mutant strain Δ*rpoE1*. The strains were cultured in XCM1 medium for 24 h and GUS activity in the total culture was determined by using ρ-nitrophenyl-β-D-glucuronide as substrate. Values given are means ± standard deviations of triplicate measurements from a representative experiment; similar results were obtained in two other independent experiments. Asterisks indicate statistically significant difference, compared with the wild type (Student's *t*-test). ^**^P < 0.01.

Global transcriptome profiling described above reveals RpoE1 positively influences the expression of the *hrp* gene cluster and related genes. The *Xcc hrp* gene cluster consists of six main operons (*hrpA-F*) (Huang et al., 2009). To validate the positive regulation of these operons by RpoE1, the expression levels of the operons in the mutant strain Δ*rpoE1* were determined by qRT-PCR. As illustrated in Figure 3A, the mRNA levels of all the *hrp* operons (*hrpA-F*) in Δ*rpoE1* cells were reduced by 76–86% compared to that in the wild-type. Two representative type III effector genes, *XC_0241* and *XC_1553*, were also examined. Their expression was also reduced by 81 and 86% in Δ*rpoE1*, respectively (Figure 3A).

In order to gain real time promoter activation data on the two representative *hrp* operons, *hrpB* and *hrpF*, and the effector genes *XC_0241* and *XC_1553* a set of GUS promotor assays were generated. To this end, a promoter-*gusA* transcriptional fusion was constructed with these target operons (or genes) (Supplementary Table S1). The resulting reporter strains were grown in XCM1 minimal medium for 24 h and GUS activity was recorded. GUS activity produced by each of the promoter-reporters in Δ*rpoE1* cells was reduced by 60–78% compared to wild type (Figure 3B). Taken together, these data indicate that the σ factor RpoE1 regulates positively the expression of *hrp* gene cluster and a large number of type III effector genes.

**Figure 4:**
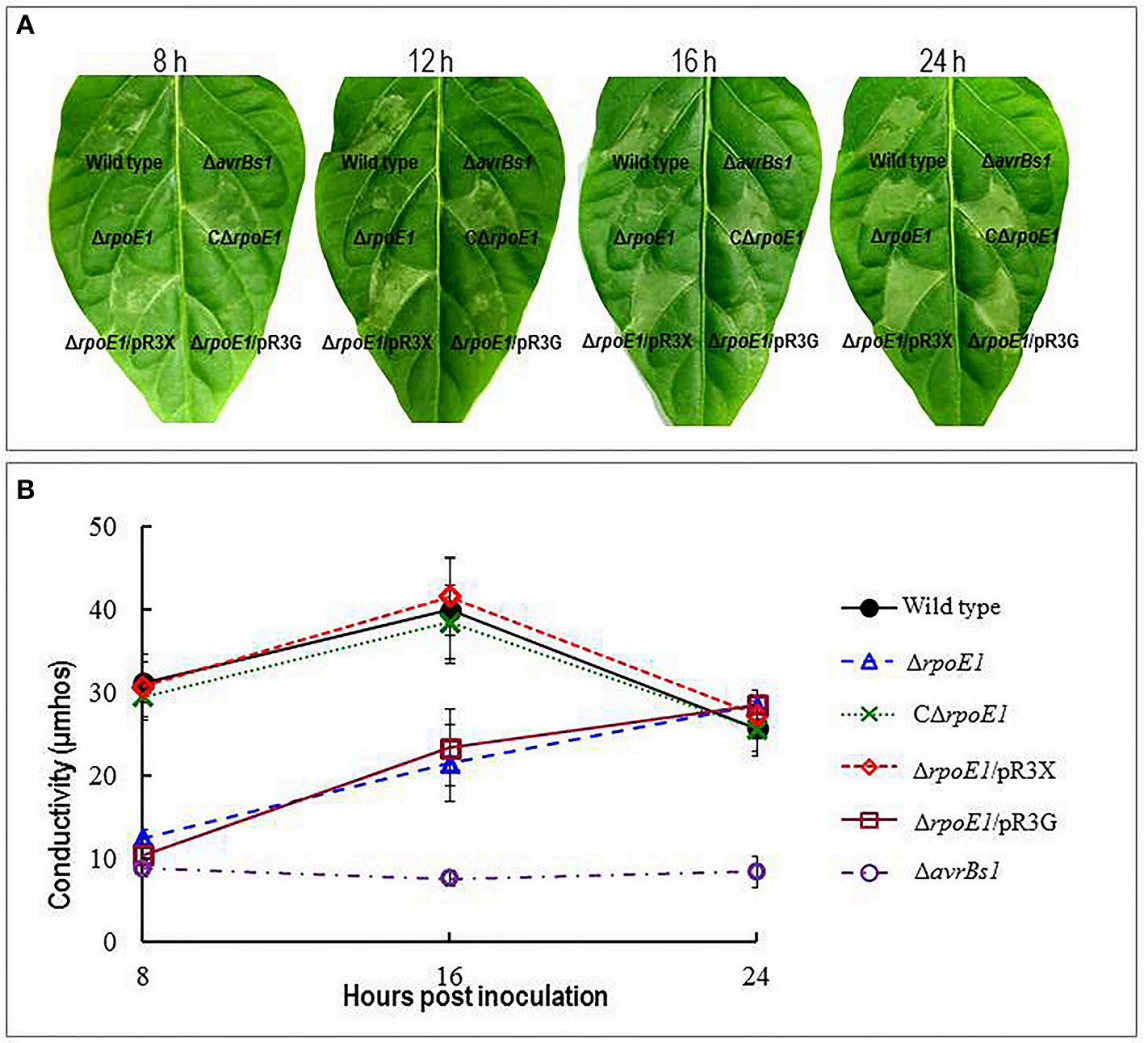
Constitutive expression of HrpX restored full HR to the mutant Δ*rpoE1*. **(A)** Bacterial cells of *Xcc* strains from overnight culture were washed and re-suspended in sterile distilled water to an OD_600_ of 0.01 (1 × 10^7^ CFU/ml). Approximately 5 μl bacterial re-suspension was infiltrated into the leaf mesophyll tissue of the non-host plant pepper ECW-10R with a blunt-end plastic syringe. Pictures of the inoculated pepper leaves were taken at 8, 12, 16, and 24 h after infiltration. **(B)** Electrolyte leakage from the pepper leaves inoculated was determined. Four 0.6 cm^2^ leaf disks for each sample were collected from the infiltrated area and incubated in 10 ml of ultrapure water. Conductivity was measured with a DDS-307A conductometer. Three samples were taken for each measurement in each experiment, and each experiment was repeated at least three times. The results presented are from a representative experiment, and similar results were obtained in all other independent experiments.

Previous studies have demonstrated that the expression of all the *hrp* genes in the *Xcc* is positively controlled by several key regulators that include HpaS, HrpG, and HrpX (Huang et al., 2009; Li et al., 2014). Global transcriptome analysis revealed that *hrpX* is one of the down-regulated genes in the Δ*rpoE1* cells grown in XCM1 minimal medium (Table 3). HrpX is an AraC-type transcriptional activator, which directly controls the expression the *hrp* operons and many type III effector genes (Koebnik et al., 2006; Huang et al., 2009). Our promoter reporter assays and qRT-PCR analyses confirmed that the expression of *hrpX* but not *hrpG* is indeed regulated positively by RpoE1 (Figure 3). These results imply that RpoE1 may regulate the expression of the *hrp* operons and the type III effector genes via HrpX. As an approach to demonstrate this idea we constitutively expressed *hrpX* in Δ*rpoE1* strain bypassing the requirement of RpoE1 for the expression of the *hrp* operons and the effector genes. This was achieved by introducing the recombinant plasmid pR3X constitutively expressing *hrpX* (Supplementary Table S1) into the *rpoE1* deletion mutant Δ*rpoE1*. The expression levels of the *hrp* operons and the effector genes *XC_0241* and *XC_1553* in the obtained recombinant strain Δ*rpoE1*/pR3X were determined by qRT-PCR. The result showed that the expression levels of all of the tested *hrp* operons and effector genes in Δ*rpoE1*/pR3X were significantly higher than those in the *rpoE1* deletion mutant Δ*rpoE1* and the wild type 8004 (Supplementary Figure S4), indicating that constitutive expression of *hrpX* could bypass RpoE1 for the expression of the genes tested. HR induction capability of strain Δ*rpoE1*/pR3X was also tested. The result displayed that the strain could produce wild-type HR symptoms (Figure 4). As expected, constitutively expressing HrpG in the mutant Δ*rpoE1* (i.e., strain Δ*rpoE1*/pR3G) could not restore HR induction (Figure 4). Taken together, the above data demonstrates that the σ^70^ factor RpoE1 influences the expression of *hrp* and type III effector genes via HrpX.

### The expression of *rpoE1* is not regulated by HrpG but is induced in minimal medium and by plant extract

**Figure 5:**
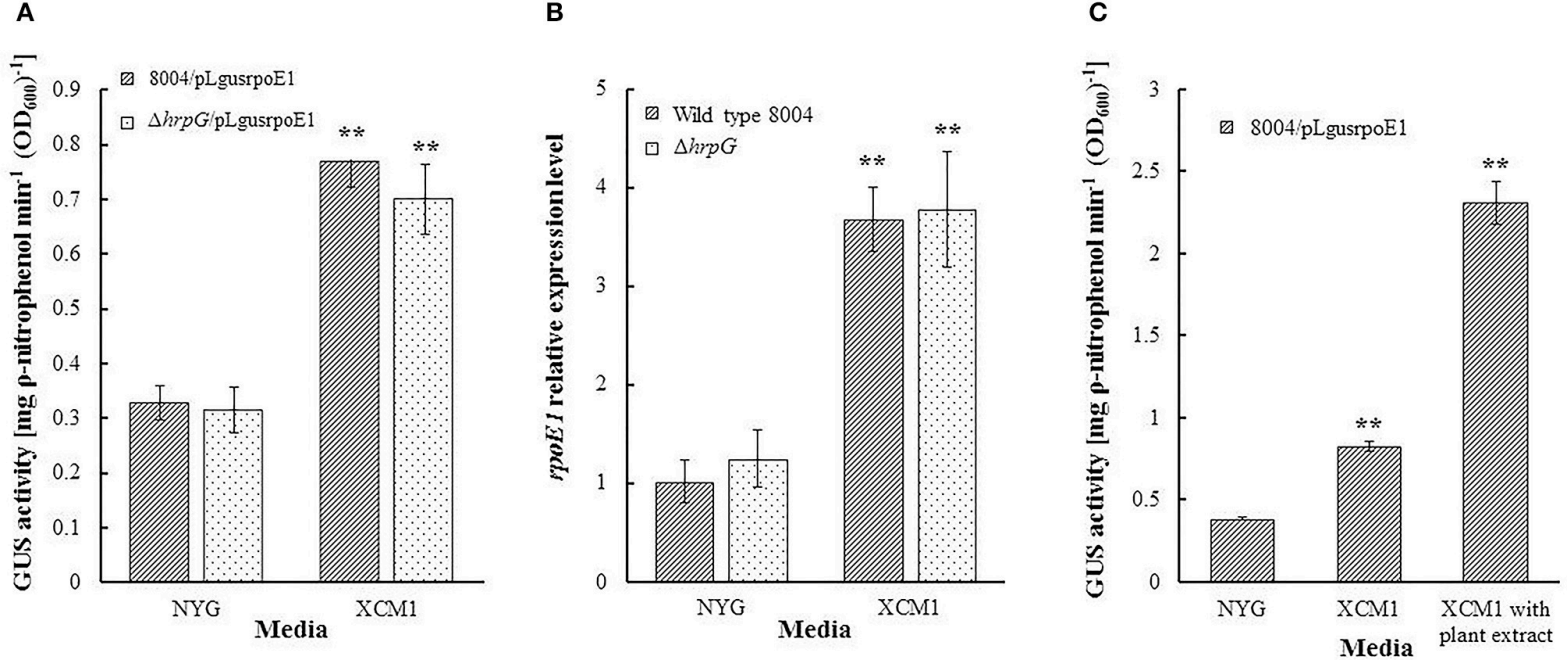
The expression of *rpoE1* is not regulated by HrpG but is induced in minimal medium and by plant extracts. The expression of *rpoE1* is not affected by deletion of HrpG but is induced in the minimal medium XCM1 compared to the nutrient rich medium NYG. The *rpoE1* promoter-*gusA* reporter plasmid pLgusrpoE1 was introduced into the wild-type strain 8004 and the *hrpG* deletion mutant Δ*hrpG* and the resulting strains 8008/pLgusrpoE1 and Δ*hrpG*/pLgusrpoE1 were grown for 24 h in NYG and XCM1, respectively. GUS activity in the total culture was determined by using ρ-nitrophenyl-β-D-glucuronide as substrate **(A)**. RNA was isolated from the cultures of 8004 and Δ*hrpG* grown in NYG and XCM1 for 24 h. The relative mRNA level of *rpoE1* was analyzed by quantitative real-time PCR and calculated with respect to the level of the corresponding transcript in the wild-type strain 8004 grown in NYG **(B)**. Strain 8004/pLgusrpoE1 was grown for 24 h in NYG, XCM1, and XCM1 supplemented with plant extract from Chinese radish leaves. GUS activity in the total culture was determined by using ρ-nitrophenyl-β-D-glucuronide as substrate **(C)**. Data are means ± standard deviations of triplicate measurements from a representative experiment; similar results were obtained in two other independent experiments. Asterisks indicate statistically significant difference, compared with other medium (Student's *t*-test). ^**^P < 0.01.

The expression of *hrpX* is positively controlled by HrpG, the OmpR-type response regulator of a two-component signal transduction system (Huang et al., 2009). Although our data suggest that RpoE1 does not directly affect *hrpG* expression, there is a possibility that HrpG may modulate the expression of *rpoE1*. To test this hypothesis, we assessed whether removal of HrpG alters the expression of *rpoE1* by promoter reporter assay. Using promoter-*gusA* transcriptional fusion reporter of *rpoE1*, named pLgusrpoE1 (Supplementary Table S1), which was constructed by fusing a DNA fragment containing the *rpoE1* promoter region to the promoterless *gusA* gene with its ribosome binding site and cloning the fused DNA segment into the vector pLAFR6. The reporter plasmid pLgusrpoE1 was introduced into the *hrpG* deletion strain (Δ*hrpG*) and the wild-type strain. These strains (Δ*hrpG*/pLgusrpoE1 and 8004/pLgusrpoE1) were grown in NYG and XCM1 media and after 24 h the GUS activities were measured. Results showed that the GUS activities produced by the two strains were similar (Figure 5A), indicating that lack of HrpG did not affect the expression of *rpoE1*. This was substantiated by qRT-PCR analysis (Figure 5B). These data indicate that HrpG does not regulate the expression of *rpoE1*. As illustrated in Figure 5B, qRT-PCR analysis showed that the expression level of *rpoE1* in the wild-type cells grown in XCM1 was almost 4 times as high as grown in NYG. Moreover, the result from the promoter reporter assay also displayed that the GUS activity produced by 8004/pLgusrpoE1 in XCM1 was twice as high as in NYG (Figure 5A). These data reveal that the expression of *rpoE1* is induced in the minimal medium XCM1. The expression of *rpoE1* was further determined in XCM1 medium supplemented with plant extract from Chinese radish leaves. As shown in Figure 5C, the GUS activity produced by 8004/pLgusrpoE1 in XCM1 supplemented with plant extract was significantly higher than that in XCM1, suggesting that the expression of *rpoE1* is stimulated in host plant.

### RpoE1 induces and modulates expression of *hrpX in vivo* and *in vitro*

**Figure 6:**
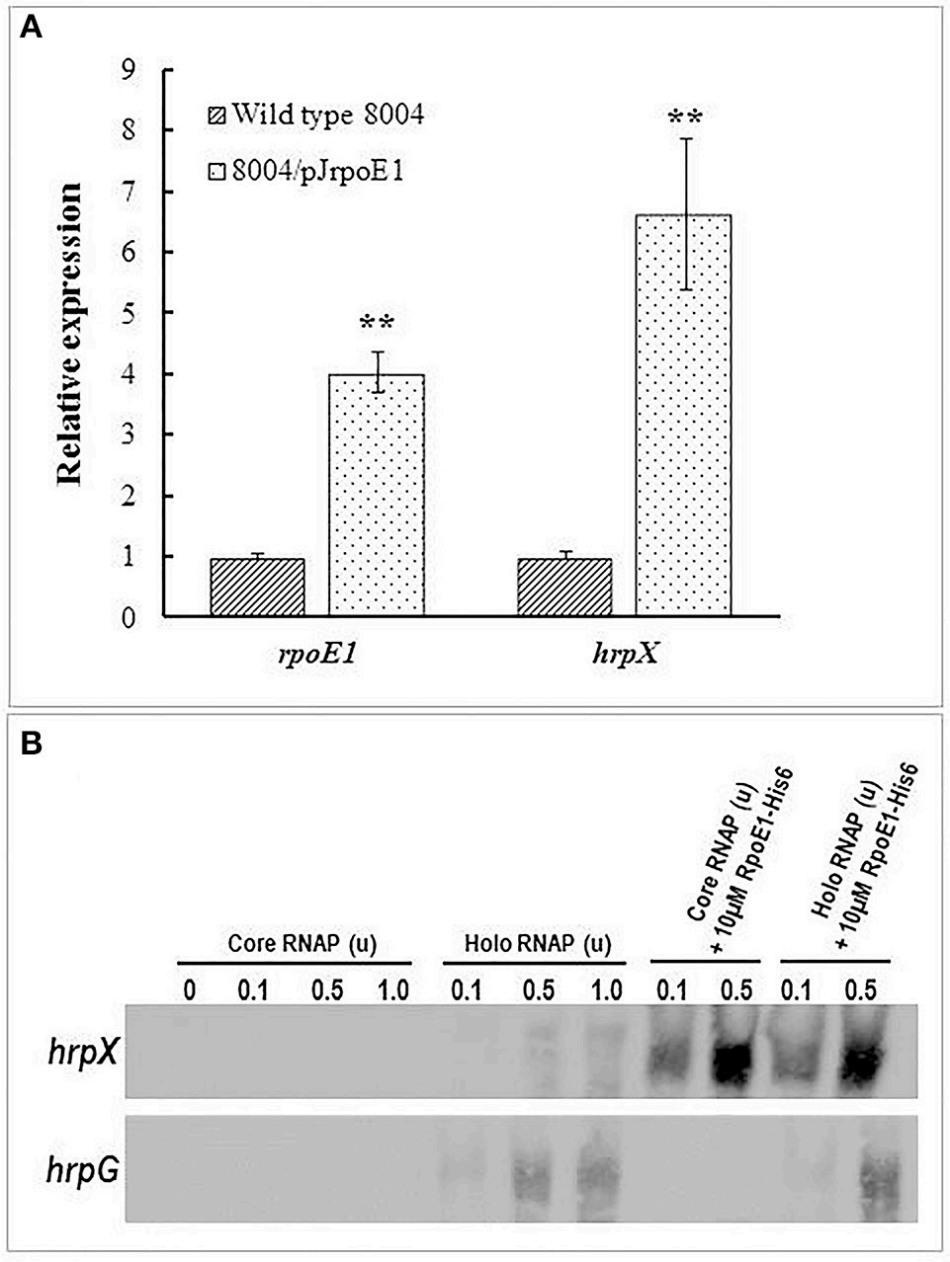
RpoE1 enhances the expression of *hrpX in vivo* and *in vitro*. **(A)** Quantitative real-time PCR (qRT-PCR) showed that overexpression of RpoE1 in *Xcc* enhanced *hrpX* transcription. For the qRT-PCR, *Xcc* wild-type strain 8004 and its derivative strain 8004/pJrpoE1 which overexpresses RpoE1 in the wild-type background were grown in the minimal medium XCM1. RNA was isolated from the cultures after incubation for 24 h. The relative mRNA level of *hrpX* was analyzed by qRT-PCR and calculated with respect to the transcript level in strain 8004. Data are means ± standard deviations of triplicate measurements from a representative experiment; similar results were obtained in two other independent experiments. Asterisks indicate statistically significant difference (Student's *t*-test). ^**^P < 0.01. **(B)** *In vitro* transcription analysis revealed that RpoE1-His6 together with core RNAP could promote *hrpX* transcription. The *in vitro* transcription assay was carried out by using 2 nM *hrpX* or *hrpG* promoter-containing template DNA and certain amount (unit) of *E. coli* core or holo RNA polymerase (core or holo RNAP).

Our qRT-PCR and promoter reporter analyses demonstrated that *hrpX* is still expressed at a certain level in the *rpoE1* deletion mutant background (Figure 3) suggesting that RpoE1 is not an essential factor but an enhancer for *hrpX* expression activity. In order to gain evidence to support this extrapolation, *rpoE1* was overexpressed in wild-type strain and the expression level of *hrpX* gene was assessed. For this purpose, the promoterless *rpoE1* was cloned into the vector pLAFRJ in an orientation allowing *rpoE1* to be driven by the *lac* promoter and the resulting recombinant plasmid named pJrpoE1 was introduced into the wild-type. The results from qRT-PCR analysis showed that expression level of *hrpX* was enhanced approximately six times in the *rpoE1* overexpressing strain (8004/pJrpoE1), compared to the wild type (Figure 6A). This indicates that RpoE1 could enhance *hrpX* expression *in vivo*.

*In vitro* evidence was also acquired using a 6 × His-tagged RpoE1 protein (RpoE1-His6). This protein was constructed, overexpressed, and purified from *E. coli*. Using the RpoE1-His6 protein an *in vitro* transcription assay was carried out where the 647 bp template DNA fragment extending from −438 to +209 relative to the TIS (transcription initiation site) of *Xcc hrpX* gene and the *E. coli* RNAP holoenzyme ($\alpha_{2}\beta \beta^{\prime }\omega \sigma^{70}$) or core enzyme ($\alpha_{2}\beta \beta^{\prime }\omega$) were used. The result showed that for the RNAP holoenzyme a certain amount of *hrpX* transcripts could be generated without RpoE1-His6 protein; however, the *hrpX* transcript level was significantly increased when RpoE1-His6 protein was added to the reaction mixture, even in the case with RNAP core enzyme only (Figure 6B). The data suggest that RpoE1-His6 could enhance significantly *hrpX* transcription *in vitro*. As expected, RpoE1-His6 did not have an effect on *in vitro* transcription of *hrpG* (Figure 6B).

### RpoE1 binds to the *hrpX* promoter region *in vivo* and *in vitro* to modulate gene transcription

As discussed above, bacterial σ^70^ factors activate gene transcription by binding to the promoter region of target genes (Kazmierczak et al., 2005; Davis et al., 2017). To confirm that RpoE1 regulates directly the expression of *hrp*/*hrc*/*hpa* and type III effector genes by binding the promotor of *hrpX* we carried out several *in vivo* and *in vitro* assays.

**Figure 7:**
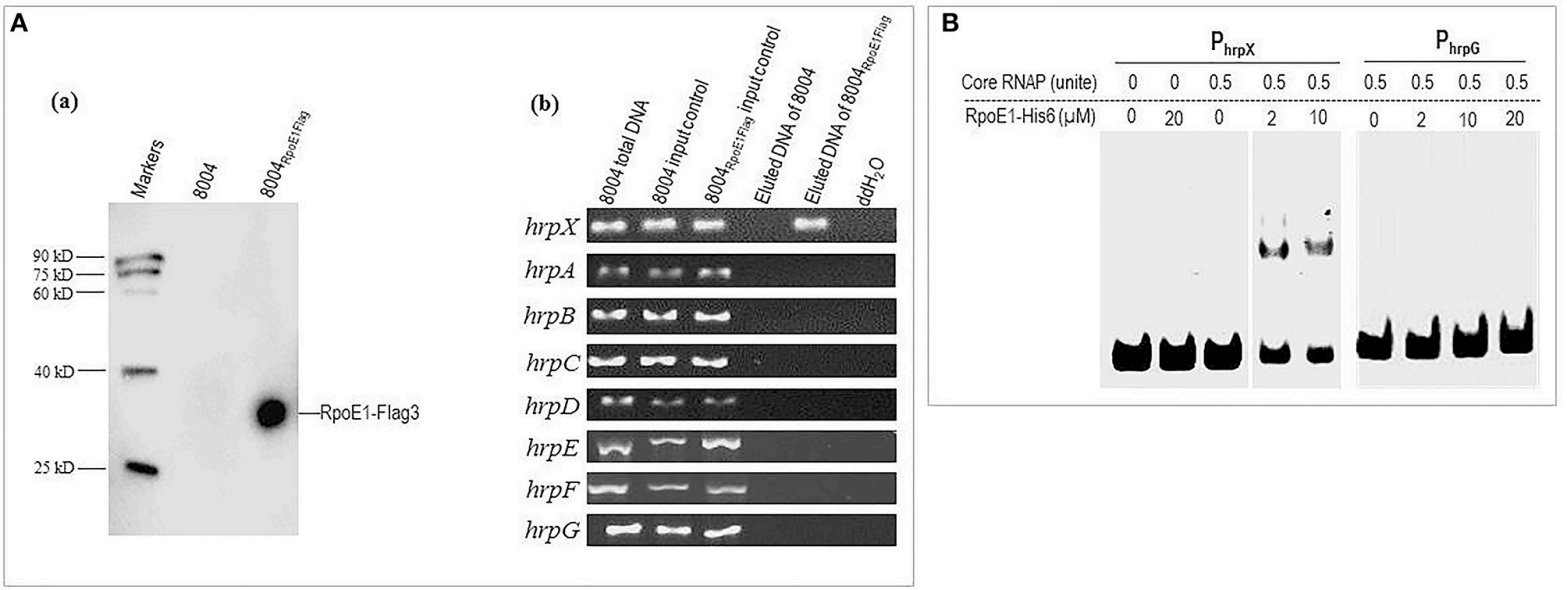
RpoE1 binds to the *hrpX* promoter region *in vivo* and *in vitro*. **(A)** ChIP assay showed that RpoE1-Flag3 could bind to the *hrpX* promoter region. (a) Western blotting of the eluted RpoE1-Flag3 protein in the ChIP assay. (b) PCR detection of eluted DNA. **(B)** EMSA result showed that RpoE1-His6 could interact with *hrpX* promoter region. A FAM-labeled 354 bp *hrpX* promoter-containing DNA fragment (P_hrpX_) was incubated with certain amount (unit) of *E. coli* core RNA polymerase (core RNAP) and certain amount (μM) of purified RpoE1-His6 for 20 min at room temperature. A FAM-labeled 353 bp *hrpG* promoter-containing DNA fragment (P_hrpG_) was used as a control.

Initially, an *in vivo* RpoE1 protein-*hrpX* promoter DNA complex assay was carried out by ChIP assay. A wild-type strain expressing the RpoE1-Flag3 (8004_RpoE1Flag_) was generated (Supplementary Table S1), for this a DNA segment encoding 3×Flag tag was fused to the 3′ end of the *rpoE1* gene in the genome of the wild-type. The resulting strains were grown in the minimal medium XCM1 for 24 h and used for the ChIP assay. A Western blot assay showed that the 3×Flag fused RpoE1 protein (RpoE1-Flag3) could be eluted from the sample strain 8004_RpoE1Flag_ but not the wild-type strain 8004 (Figure 7A). As illustrated in Figure 7A, the result of ChIP assay showed that using the eluted DNA from RpoE1-Flag3 protein as template, PCR product was obtained by the primer pair designed for amplification of DNA fragment containing *hrpX* promoter, but no product could be obtained by the primers for *hrpG* or *hrpA-F* operons, suggesting that RpoE1 binds directly to *hrpX* promoter region *in vivo*.

For *in vitro* determination of whether RpoE1 interacts with the *hrpX* promoter an EMSA was carried out. Here, a 354-bp DNA fragment containing the *hrpX* promoter region from 294 bp upstream to 60 bp downstream of *hrpX* translational start codon was amplified by the FAM-labeled primers and designated as P_hrpX_. The binding ability of RpoE1-His6 to P_hrpX_ was then determined by EMSA. A 353 bp FAM-labeled fragment containing *hrpG* promoter, named P_hrpG_, was also obtained and included in the EMSA experiment. As shown in Figure 7B, the RpoE1-His6 protein alone did not interact with P_hrpX_; however, it could bind P_hrpX_ and retarded P_hrpX_ to a defined position when the core RNAP was added. No interaction between RpoE1-His6 and P_hrpG_ was observed in the experiment (Figure 7B). This suggests that the binding of RpoE1-His6 to the *hrpX* promoter was specific. Taken together, these data suggest that RpoE1 directly interacts with the *hrpX* promoter region.

## Discussion

Here, we report the systematic characterization of σ factors that are involved in virulence regulation in the phyotpathogen *Xcc*. This work led to the identification of important roles in *Xcc* survival and pathogenesis for previously uncharacterized *Xcc* σ factors including the alternative σ^70^ factor RpoE1 and the role it plays in virulence and the regulation of T3SS.

The *Xcc* strain 8004 possesses 15 ORFs encoding two putative σ^54^ and 13 σ^70^ factors (Table 1). Our bioinformatic analysis revealed that these ORFs are highly conserved in the other two completely sequenced *Xcc* genomes, i.e., ATCC33913, and B100 (da Silva et al., 2002; Vorhölter et al., 2008). Moreover, the 13 predicted σ^70^ family members could be assigned to group I (1 identified), III (2 identified), and IV (10 identified). The group I member encoded by *XC_3806* was predicted as the housekeeping σ factor RpoD that is indispensable for the expression of essential genes. Consequently, no mutant strain could be generated for *XC_3806* (*rpoD*). Similarly, no mutant strain could be created for *XC_3843*, a gene encoding a σ^70^ group III member homologous to RpoH, which was found to be essential σ factor for the growth of *E. coli* and *Francisella tularensis* (Zhou et al., 1988; Grall et al., 2009). This work showed that when an extra copy of *XC_3843* (*rpoH*) was introduced into *Xcc* cells *in trans*, the chromosomal *XC_3843* (*rpoH*) could be removed from the cells. Similarly, a mutation of *XC_3806* (*rpoD*) could be generated in the same way. These data provide evidence indicating that both RpoD and RpoH are essential σ factors for *Xcc* growth in the conditions tested (in the nutrient rich medium NYG and the optimum growth temperature 28°C). As shown in Table 1, RpoD possesses four domains named σ_1_, σ_2_, σ_3_, and σ_4_, while RpoH has only σ_2_, σ_4_, and partial σ_1_ domains. It is believed that a Gram-negative bacterium have over 300 essential genes that are critical for survival and growth (Juhas et al., 2014). Structurally different RpoD and RpoH are central to growth raises a lot of questions that would be interesting to address going forward. These would include asking the questions: Do RpoD and RpoH control all the essential genes in *Xcc* together or they control subsets of essential genes independently? If they work together, how do they regulate the expression of the target genes? Notably, homologs of these two σ factors are present not only in all other *Xanthomonas* species but also in most plant associated species of *Pseudomonas, Ralstonia*, and *Erwinia* (Table 2). This suggests that this mechanism for viability control is present in these strains but it would be interesting to know whether they are essential for these strains also.

The remaining predicted σ factor-encoding genes could be individually deleted from the genome of *Xcc* strain 8004. Using these mutants in leaf clipping assays revealed that a strain deficient in the σ^70^ factor RpoE1, had attenuated virulence toward the host plant Chinese radish but the strains defective for the other σ factors displayed virulence similar to that of the wild-type (Figure 1). RpoE1 is one of the 10 predicted σ^70^ family group IV members. Deletion of all of the other nine group IV members, i.e., mutant strain Δ9, did not alter the virulence of *Xcc* (Figure 1). Furthermore, the mutant strain Δ10, in which all of the 10 group IV members were deleted, had similar virulence to the *rpoE1* deletion mutant Δ*rpoE1* (Figure 1). These data suggest that all of the predicted σ^70^ family factors except RpoE1 are not involved in *Xcc* virulence in the tested conditions. Notably, unlike many other bacteria which encode only one σ^54^ factor, the overwhelming majority of xanthomonads harbor two σ^54^ factors (RpoN1 and RpoN2). To date, the whole genome sequences of 28 *Xanthomonas* species or pathovars are available and 26 of them have two σ^54^ factors (https://www.ncbi.nlm.nih.gov/genome/?term=xanthomonas). In addition to xanthomonads, some other bacteria also contain more than one σ^54^ factors. For instance, *Ralstonia solanacearum* and *Rhodobacter sphaeroides* have two and four σ^54^ factors, respectively (Domenzain et al., 2012; Ray et al., 2015). It has been shown that RpoN1, but not RpoN2, is required for twitching motility, natural competence, growth on nitrate, and virulence of *Ralstonia solanacearum* (Ray et al., 2015). In *X. oryzae* pv. *oryzae* RpoN2 is required for flagellar motility and full virulence (Tian et al., 2015). Both RpoN1 and RpoN2 contribute to the virulence of *X. citri* subsp. *citri* (Gicharu et al., 2016). Interestingly, the *rpoN1* mutant of *X*. *citri* subsp. *citri* showed a reduction in cell motility, while the *rpoN2* mutant increased cell motility, suggesting that the RpoN1 and RpoN2 play diverse roles in *X. citri* subsp. *citri* (Gicharu et al., 2016). Our data presented here showed that the mutant strain Δ*rpoN1rpoN2* defective in both σ^54^ factors still had wild-type virulence (Figure 1), suggesting that the σ^54^ factors are not involved in *Xcc* virulence. Similarly, previous work demonstrated that one of the *rpoN* genes is not required for the pathogenicity of *X. campestris* pv. *vesicatoria* (Horns and Bonas, 1996). Yang et al. (2009) showed that the RpoN2 of *Xcc* is essential for motility and normal flagellar biogenesis. It is worthy to investigate whether the σ^54^ factors in *Xcc* are involved in any other cellular processes in addition to flagellar biogenesis. Only the mutation of *rpoE1* resulted in a significant reduction in pathogenicity and HR of *Xcc* in plants. This was mirrored by gene expression analysis revealing that RpoE1 had a role to play in controlling the expression of the *hrp* gene cluster and therefore regulating positively the T3SS. It is well known that the T3SS is crucial for the pathogenicity of many Gram-negative bacterial pathogens, including a large number of important plant pathogens in the genera *Erwinia, Pantoea, Pectobacterium, Pseudomonas, Ralstonia* as well as *Xanthomonas* (He, 1998; Galán and Collmer, 1999; Cornelis and Van Gijsegem, 2000; Galán and Wolf-Watz, 2006). The T3SS systems of these pathogens are encoded by the *hrp* cluster of genes which is highly conserved and acquired by horizontal gene transfer (Gürlebeck et al., 2006). However, it is believed that due to evolution, the pathogens have formed two groups based on their manner of *hrp* gene transcriptional regulation (Tang et al., 2006; Mole et al., 2007). The first group (Group A) includes the pathogens in the genera *Erwinia, Pantoea, Pectobacterium*, and *Pseudomonas*, while the second group (Group B) is composed of the pathogens of the genera *Ralstonia* and *Xanthomonas*. The expression of the *hrp* gene cluster in Group A is directly activated by the alternative σ^70^ factor named HrpL, while in Group B is controlled by AraC-type transcriptional regulator named HrpX (for *Xanthomonas*) or HrpB (for *Ralstonia*) (Tang et al., 2006; Mole et al., 2007). It has been demonstrated that the expression of *hrpX* is positively regulated by the two-component signal transduction system composed of the sensor histidine kinase HpaS and the OmpR-type response regulator HrpG (Büttner and Bonas, 2010; Li et al., 2014). Transcriptome, qRT-PCR, and promoter reporter analyses showed that mutation of *rpoE1* reduced the expression of *hrpX* (but not *hrpG*) (Table 3, Figure 3). Moreover, mutation of *hrpG* did not alter *rpoE1* expression (Figure 5). These data imply that there is no regulatory relationship between HrpG and RpoE1 at transcriptional level. However, RpoE1 and HrpG may regulate the expression of *hrpX* independently of each other. Notably, a comparison revealed that RpoE1 and the HrpL of the Group A pathogens share only about 23% identity in amino acid sequences. ChIP analysis showed that unlike HrpL which directly regulates the *hrp* cluster of genes RpoE1 does not target any operons of the *hrp* gene cluster (Figure 7A). These data suggest that RpoE1 and HrpL inference the expression of *hrp* genes via different manners. Furthermore, as shown in Table 2, *Xcc* does not have a σ^70^ factor highly homologous to HrpL, but the Group A pathogens such as *Pseudomonas syringae* pv. *tomato* and *Erwinia amylovora* harbor a RpoE highly homologous to the RpoE1 of *Xcc*. It would be interesting to know whether this RpoE is involved in the *hrp* gene regulation of these pathogens.

Our ChIP and EMSA analyses revealed that RpoE1-His6 could bind to the promoter region of *hrpX in vivo* and *in vitro* (Figure 7), suggesting that RpoE1 regulates directly the expression of *hrpX*. *In vitro* transcription assay displayed that RpoE1-His6 could enhance the *E. coli* holo RNAP-initiated *hrpX* transcription (Figure 6A). More importantly, RpoE1-His6 together with the core RNAP could promote the transcription of *hrpX* (Figure 6B), suggesting that RpoE1 may act as the σ factor that composes the holo RNAP directing *hrpX* transcription in *Xcc*. Furthermore, bioinformatics analysis revealed that RpoE1 is a member of ECF σ^70^ factors that normally respond to environmental stresses. This together with the fact that the expression of *rpoE1* is induced *in planta* suggests that the RpoE1-associated regulation of *hrpX* may be related to an undefined plant signal. Work to identify the signal and the mechanism by which it controls RpoE1 will be the subject for further studies. Interestingly, the homologs of RpoE1 are widely distributed in other *Xanthomonas* species as well as *Pseudomonas, Ralstonia*, and *Erwinia* strains suggesting a similar mechanism might be broadly conserved in many plant associated bacteria.

## Author contributions

J-LT and B-LJ conceived the study. L-YY, L-CY, and Y-LG carried out the experiments. LW, W-ZZ, Y-QH, and WJ performed bioinformatics analysis. J-LT and B-LJ wrote the manuscript.

### Conflict of interest statement

The authors declare that the research was conducted in the absence of any commercial or financial relationships that could be construed as a potential conflict of interest.

## Supplementary material

Supplementary Figure S1 PCR detection showing that the chromosomal *XC_3806* or *XC_3843* could be deleted from the *Xcc* cells harboring an extra copy of *XC_3806* or *XC_3843 in trans*. *XC_3806* and *XC_3843* in the recombinant strains 8004/pJ3806 and 8004/pJ3843 were deleted respectively by double-crossover homologous recombination method using the suicide plasmid pK18mobsacB as a vector. Three mutant colonies for each strain were randomly selected for PCR verification by the primer sets D3806LF/D3806RR and D3843LF/D3843RR complemented respectively to the flanking regions of *XC_3806* and *XC_3843* in the chromosome of strain 8004 but not any sequence in the plasmids pJ3806 and pJ3843. **(A)** PCR verification showing that the chromosomal *XC_3806* was deleted. Lane 1: PCR product, using the total DNA of 8004 as template, with expected size of 3379 bp; lanes 2-4: PCR products, using the total DNA of selected mutant colonies as templates, with expected size of 1511 bp, suggesting that *XC_3806* was removed. **(B)** PCR verification showing that the chromosomal *XC_3843* was deleted. Lane 1: PCR product, using the total DNA of 8004 as template, with expected size of 2272 bp; lanes 2-4: PCR products, using the total DNA of selected mutant colonies as templates, with expected size of 1404 bp, suggesting that *XC_3843* was removed.

Supplementary Figure S2 The mutant strain Δ*rpoE1* and the wild type strain 8004 had a similar growth rate. The *Xcc* strains were incubated in the nutrient rich medium NYG and the minimal medium XCM1. The optical density at 600 nm (OD_600_) of the cultures was measured at 4 h intervals.

Supplementary Figure S3 The production of the extracellular amylase, endoglucanase, protease, and exopolysaccharide produced by the mutant strains Δ*rpoE1* and Δ10 is no significantly different compared to the wild-type strain 8004. Exopolysaccharide production and amylase, endoglucanase, and protease activity were detected by growing bacterial cells at 28°C on NYG plates supplemented with 2% (wt/vol) glucose, 0.1% (wt/vol) starch, 0.25% (wt/vol) carboxymethycellulose, and 0.5% (wt/vol) skimmed milk, respectively. For amylase and endoglucanase, the plates were stained after incubation for 24 h and photographed. The plates for exopolysaccharide production and protease activity were photographed after incubation for 48 h. The clear zones created by the degradation of the substrates and the sticky gum in the colonies reflect the enzyme activities and exopolysaccharide production, respectively. The experiment was repeated three times and the results were similar.

Supplementary Figure S4 Constitutive expression of *hrpX* could bypass the requirement of RpoE1 for the expression of the *hrp* and type III effector genes, revealed by quantitative real-time PCR (qRT-PCR). RNAs were isolated from the cells of *Xcc* strains grown in the minimal medium XCM1 for 24 h. The relative mRNA levels of the *hrp* gene cluster (*hrpA*-*F*), the master *hrp* regulator *hrpX*, and the type III effector-encoding genes *XC_0241* and *XC_1553* were analyzed by quantitative real-time PCR and calculated with respect to the transcript level in the wild-type strain 8004. Data are means ± standard deviations of triplicate measurements from a representative experiment; similar results were obtained in two other independent experiments. Wild type, the wild-type strain 8004; Δ*rpoE1*, the *rpoE1* deletion mutant strain; Δ*rpoE1*/pR3X, Δ*rpoE1* constitutively expressing *hrpX*.

Supplementary Table S1 Bacterial strains and plasmids used in this work.

Supplementary Table S2 Primers used in this study.

Supplementary Table S3 Statistics analysis of the virulence of *Xcc* strains.
