# Supplementary figures and images for: Systematic Functional Analysis of Sigma (σ) Factors in the Phytopathogen Xanthomonas campestris Reveals Novel Roles in the Regulation of Virulence and Viability
Source: Front Microbiol. 2018 Aug 3;9:1749. doi: 10.3389/fmicb.2018.01749 (PMC6085468; doi:10.3389/fmicb.2018.01749)

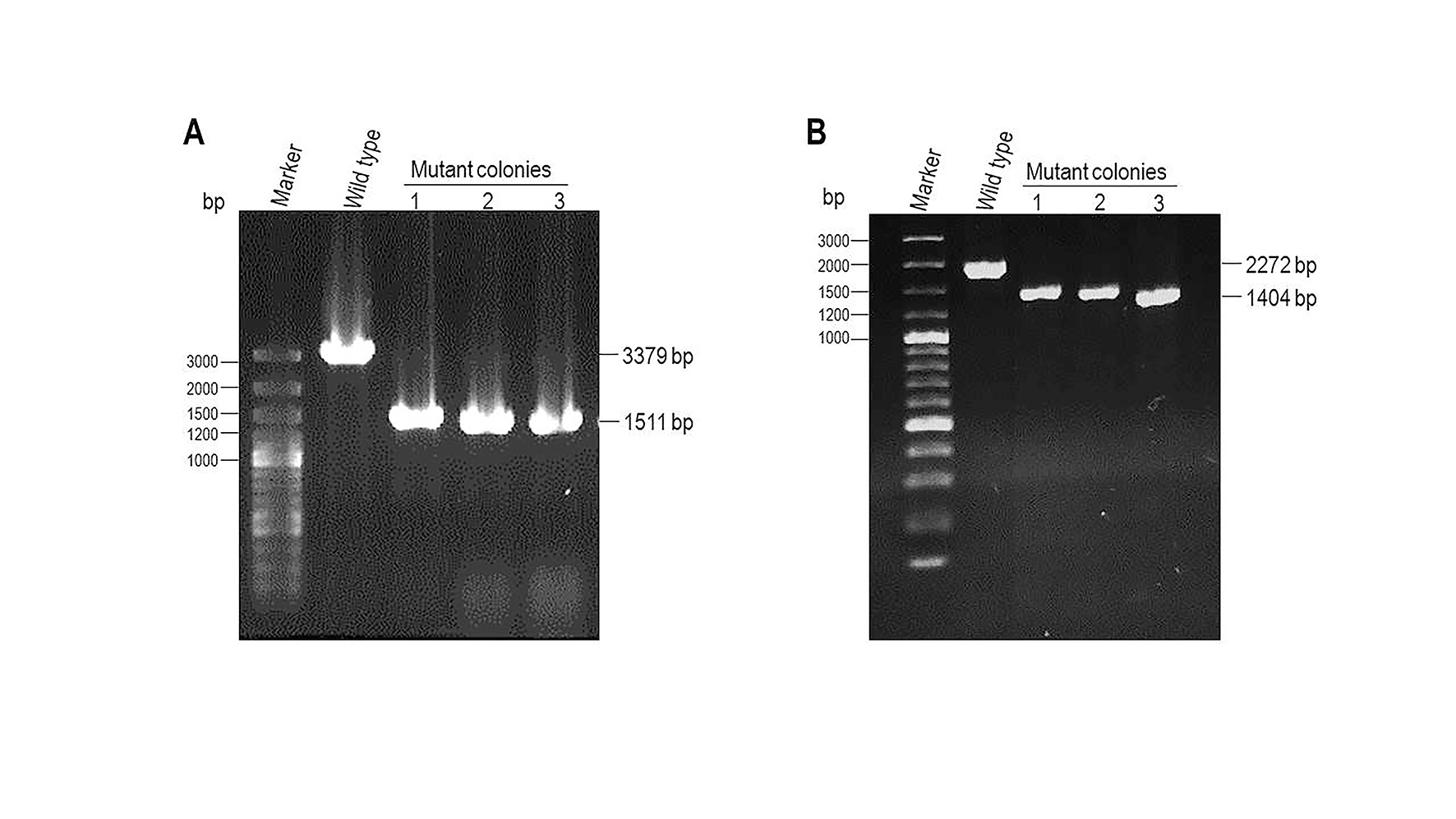

Supplement: Supplementary Figure S1 — PCR detection showing that the chromosomal XC_3806 or XC_3843 could be deleted from the Xcc cells harboring an extra copy of XC_3806 or XC_3843 in trans. XC_3806 and XC_3843 in the recombinant strains 8004/pJ3806 and 8004/pJ3843 were deleted respectively by double-crossover homologous recombination method using the suicide plasmid pK18mobsacB as a vector. Three mutant colonies for each strain were randomly selected for PCR verification by the primer sets D3806LF/D3806RR and D3843LF/D3843RR complemented respectively to the flanking regions of XC_3806 and XC_3843 in the chromosome of strain 8004 but not any sequence in the plasmids pJ3806 and pJ3843. (A) PCR verification showing that the chromosomal XC_3806 was deleted. Lane 1: PCR product, using the total DNA of 8004 as template, with expected size of 3379 bp; lanes 2-4: PCR products, using the total DNA of selected mutant colonies as templates, with expected size of 1511 bp, suggesting that XC_3806 was removed. (B) PCR verification showing that the chromosomal XC_3843 was deleted. Lane 1: PCR product, using the total DNA of 8004 as template, with expected size of 2272 bp; lanes 2-4: PCR products, using the total DNA of selected mutant colonies as templates, with expected size of 1404 bp, suggesting that XC_3843 was removed. [file Image_1.JPEG]
