# Supplementary material for: Systematic Functional Analysis of Sigma (σ) Factors in the Phytopathogen Xanthomonas campestris Reveals Novel Roles in the Regulation of Virulence and Viability
Source: Front Microbiol. 2018 Aug 3;9:1749. doi: 10.3389/fmicb.2018.01749 (PMC6085468; doi:10.3389/fmicb.2018.01749)
Supplement: Supplementary Table S1 — Bacterial strains and plasmids used in this work. [file Table_1.DOCX]

**Table S1. Bacterial strains and plasmids used in this work**

| Strains or plasmids | Relevant characteristics^a^ | Reference or source |
| --- | --- | --- |
| *Escherichia coli* |  |  |
| DH5α | F^-^ Φ80*lac*ZΔM15Δ(*lac*ZYA-*arg*F) U169 *rec*A1 *end*A1 *hsd*R17(r_k_^-^,m_k_^+^) *pho*A *sup*E44 *thi*-1 *gyr*A96 *rel*A1 λ^-^ | Gibco BRL, Life Technologies |
| BL21(DE3) | F^-^ *ompT hsdS_B_* (*r_B_^-^*, *m_B_^-^*) *gal dcm* (DE3) | Novagen |
| BL21(DE3)/pET2974 | BL21(DE3) harboring pET2974, Kan^r^ | This work |
|  |  |  |
| *Xcc* strains |  |  |
| *Xcc* 8004 | Wild type, Rif^r^ | Qian et al., 2005 |
| Δ*avrBs1* | Deletion mutant of *avrBs1*, Rif^r^ | Xu et al., 2008 |
| Δ*hrpG* | Deletion mutant of *hrpG*, Rif^r^ | This work |
| Δ*rpoE1* | Deletion mutant of *XC_2974*, Rif^r^ | This work |
| CΔ*rpoE1* | Cis-complementation of Δ*rpoE1*, Rif^r^ | This work |
| Δ*rpoE2* | Deletion mutant of *XC_3383*, Rif^r^ | This work |
| Δ*rpoE3* | Deletion mutant of *XC_2905*, Rif^r^ | This work |
| Δ*rpoE4* | Deletion mutant of *XC_2566*, Rif^r^ | This work |
| Δ*rpoE5* | Deletion mutant of *XC_1474*, Rif^r^ | This work |
| Δ*rpoE6* | Deletion mutant of *XC_1193*, Rif^r^ | This work |
| Δ*rpoE7* | Deletion mutant of *XC_2934*, Rif^r^ | This work |
| Δ*rpoE8* | Deletion mutant of *XC_3864*, Rif^r^ | This work |
| Δ*rpoE9* | Deletion mutant of *XC_0556*, Rif^r^ | This work |
| Δ*rpoE10* | Deletion mutant of *XC_3099*, Rif^r^ | This work |
| Δ*fliA* | Deletion mutant of *XC_2281*, Rif^r^ | This work |
| Δ*rpoN1* | Deletion mutant of *XC_1311*, Rif^r^ | This work |
| Δ*rpoN2* | Deletion mutant of *XC_2251*, Rif^r^ | This work |
| Δ*rpoN1rpoN2* | Double deletion mutant of *XC_1311* and *XC_2251*, Rif^r^ | This work |
| Δ9 | Deletion mutant of the nine ECF sigma factors except *rpoE1*, Rif^r^ | This work |
| Δ10 | Deletion mutant of the ten ECF sigma factors, Rif^r^ | This work |
| Δ11 | Deletion mutant of the eleven sigma70 factors, Rif^r^ | This work |
| 8004/pJC3806 | 8004 harboring pJC3806, Rif^r^, Tc^r^ | This work |
| 8004/pJC3843 | 8004 harboring pJC3843, Rif^r^, Tc^r^ | This work |
| 8004/pLgushrpG | 8004 harboring pLgushrpG, Rif^r^, Tc^r^ | This work |
| 8004/pLgushrpX | 8004 harboring pLgushrpX, Rif^r^, Tc^r^ | This work |
| 8004/pLgushrpF | 8004 harboring pLgushrpF, Rif^r^, Tc^r^ | This work |
| 8004/pLgushrpB | 8004 harboring pLgushrpB, Rif^r^, Tc^r^ | This work |
| 8004/pLgus0241 | 8004 harboring pLgus0241, Rif^r^, Tc^r^ | This work |
| 8004/pLgus1553 | 8004 harboring pLgus1553, Rif^r^, Tc^r^ | This work |
| Δ*rpoE1*/pLgushrpG | Δ*rpoE1* harboring pLgushrpG, Rif^r^, Tc^r^ | This work |
| Δ*rpoE1*/pLgushrpX | Δ*rpoE1* harboring pLgushrpX, Rif^r^, Tc^r^ | This work |
| Δ*rpoE1*/pLgushrpF | Δ*rpoE1* harboring pLgushrpF, Rif^r^, Tc^r^ | This work |
| Δ*rpoE1*/pLgushrpB | Δ*rpoE1* harboring pLgushrpB, Rif^r^, Tc^r^ | This work |
| Δ*rpoE1*/pLgus0241 | Δ*rpoE1* harboring pLgus0241, Rif^r^, Tc^r^ | This work |
| Δ*rpoE1*/pLgus1553 | Δ*rpoE1* harboring pLgus1553, Rif^r^, Tc^r^ | This work |
| Δ*rpoE1*/pR3G | Δ*rpoE1* harboring pR3G, Rif^r^, Tc^r^ | This work |
| Δ*rpoE1*/pR3X | Δ*rpoE1* harboring pR3X, Rif^r^, Tc^r^ | This work |
| 8004/pLgusrpoE1 | 8004 harboring pLgusrpoE1, Rif^r^, Tc^r^ | This work |
| Δ*hrpG*/pLgusrpoE1 | Δ*hrpG* harboring pLgusrpoE1, Rif^r^, Tc^r^ | This work |
| 8004/pJrpoE1 | 8004 harboring pJrpoE1, Rif^r^, Tc^r^ | This work |
| 8004_RpoE1Flag_ | As *Xcc* 8004, the sequence of RpoE1 was replaced with *rpoE1*-3×*flag* on chromosome, Rif^r^ | This work |
|  |  |  |
| Plasmids |  |  |
| pK18mob | Suicide plasmid in *X. campestris* pv. *campestris*, Kan^r^ | Schäfer et al., 1994 |
| pK18mobsacB | Sucrose-sensitive suicide plasmid in *X. campestris* pv. *campestris*, Kan^r^ | Schäfer et al., 1994 |
| pLgus | pLAFR6 containing a 1,832 bp *gusA* ORF (excluding ATG), Tc^r^ | Jiang et al., 2008 |
| pLAFRJ | Shuttle plasmid pLAFR3 derivate containing the multiple cloning sites of pUC19, Tc^r^ | Jiang et al., 2009 |
| pR3G | pLAFR3 containing a fragment including promoterless *hrpG* gene, Tc^r^ | Huang et al., 2009 |
| pR3X | pLAFR3 containing a fragment including promoterless *hrpX* gene, Tc^r^ | Huang et al., 2009 |
| pET30a | Expression vector, N-terminal 6×His-tagged sequences, Kan^r^ | Novagen |
| pLgushrpG | pLAFR6 containing a *hrpG* promoter-*gusA* fusion fragment, Tc^r^ | Laboratory collection |
| pLgushrpB | pLAFR6 containing a *hrpB* promoter-*gusA* fusion fragment, Tc^r^ | Laboratory collection |
| pLgushrpF | pLAFR6 containing a *hrpF* promoter-*gusA* fusion fragment, Tc^r^ | Laboratory collection |
| pLgus0241 | pLAFR6 containing a *XC_0241* promoter-*gusA* fusion fragment, Tc^r^ | Laboratory collection |
| pLgus1553 | pLAFR6 containing a *XC_1553* promoter-*gusA* fusion fragment, Tc^r^ | Laboratory collection |
| pK2974D | pK18mobsacB containing fragments flanking *rpoE1*, Kan^r^ | This work |
| pK2974CC | pK18mobsacB containing fragments flanking *XC_0742* and promoter and ORF of *rpoE1* fragment, Kan^r^ | This work |
| pK3383D | pK18mobsacB containing fragments flanking *rpoE2*, Kan^r^ | This work |
| pK2905D | pK18mobsacB containing fragments flanking *rpoE3*, Kan^r^ | This work |
| pK2566D | pK18mobsacB containing fragments flanking *rpoE4*, Kan^r^ | This work |
| pK1474D | pK18mobsacB containing fragments flanking *rpoE5*, Kan^r^ | This work |
| pK1193D | pK18mobsacB containing fragments flanking *rpoE6*, Kan^r^ | This work |
| pK2934D | pK18mobsacB containing fragments flanking *rpoE7*, Kan^r^ | This work |
| pK3864D | pK18mobsacB containing fragments flanking *rpoE8*, Kan^r^ | This work |
| pK0556D | pK18mobsacB containing fragments flanking *rpoE9*, Kan^r^ | This work |
| pK3099D | pK18mobsacB containing fragments flanking *rpoE10*, Kan^r^ | This work |
| pK2281D | pK18mobsacB containing fragments flanking *fliA*, Kan^r^ | This work |
| pK1311D | pK18mobsacB containing fragments flanking *rpoN1*, Kan^r^ | This work |
| pK2251D | pK18mobsacB containing fragments flanking *rpoN2*, Kan^r^ | This work |
| pJC3843 | pLAFRJ containing a 1476 bp fragment including the *rpoH*, Tc^r^ | This work |
| pJC3806 | pLAFRJ containing a 2471 bp fragment including the *rpoD*, Tc^r^ | This work |
| pLgushrpX | pLAFR6 containing a *hrpX* promoter-*gusA* fusion fragment, Tc^r^ | This work |
| PLgusrpoE1 | pLAFR6 containing a *rpoE1* promoter-*gusA* fusion fragment, Tc^r^ | This work |
| pJrpoE1 | pLAFRJ containing a fragment including promoterless *rpoE1* gene, Tc^r^ | This work |
| pET2974 | pET30a containing a 621 bp fragment of *rpoE1* coding region, Kan^r^ | This work |
| pKrpoE1Flag | pK18mobsacB containing 3×flag and fragments flanking *rpoE1*, Kan^r^ | This work |

^a^Rif^r^, Kan^r^, and Tc^r^ indicate resistance to rifampicin, kanamycin, and tetracycline, respectively.

Huang, D.L., Tang, D.J., Liao, Q., Li, X.Q., He, Y.Q., Feng, J.X., et al. (2009). The Zur of *Xanthomonas campestris* is involved in hypersensitive response and positively regulates the expression of the *hrp* cluster via *hrpX* but not *hrpG*. *Mol. Plant-Microbe Interact.* 22, 321-329. <https://doi.org/10.1094/MPMI-22-3-0321>.

Jiang, B.L., He, Y.Q., Cen, W.J., Wei, H.Y., Jiang, G.F., Jiang, W., et al. (2008). The type III secretion effector XopXccN of *Xanthomonas campestris* pv. *campestris* is required for full virulence. *Res. Microbiol.* 159, 216-220. <https://doi.org/10.1016/j.resmic.2007.12.004>.

Jiang, W., Jiang, B.L., Xu, R.Q., Huang, J.D., Wei, H.Y., Jiang, G.F., et al. (2009). Identification of six type III effector genes with the PIP box in *Xanthomonas campestris* pv. *campestris* and five of them contribute individually to full pathogenicity. *Mol. Plant-Microbe Interact.* 22, 1401-1411. <https://doi.org/10.1094/MPMI-22-11-1401>.

Qian, W., Jia, Y., Ren, S.X., He, Y.Q., Feng, J.X., Lu, L.F., et al. (2005). Comparative and functional genomic analyses of the pathogenicity of phytopathogen *Xanthomonas campestris* pv. *campestris*. *Genome Res.* 15, 757-767. <https://doi.org/10.1101/gr.3378705>.

Schäfer, A., Tauch, A., Jäger, W., Kalinowski, J., Thierbach, G., and Pühler, A. (1994). Small mobilizable multi-purpose cloning vectors derived from the *Escherichia coli* plasmids pK18 and pK19: selection of defined deletions in the chromosome of *Corynebacterium glutamicum*. *Gene* 145, 69-73. <https://doi.org/10.1016/0378-1119(94)90324-7>.

Xu, R.Q., Blanvillain, S., Feng, J.X., Jiang, B.L., Li, X.Z., Wei, H.Y., et al. (2008). AvrAC_Xcc8004_, a type III effector with a leucine-rich repeat domain from *Xanthomonas campestris* pathovar *campestris* confers avirulence in vascular tissues of *Arabidopsis thaliana* ecotype Col-0. *J. Bacteriol.* 190, 343-355. <https://doi.org/10.1128/JB.00978-07>.
