# Supplementary material for: Systematic Functional Analysis of Sigma (σ) Factors in the Phytopathogen Xanthomonas campestris Reveals Novel Roles in the Regulation of Virulence and Viability
Source: Front Microbiol. 2018 Aug 3;9:1749. doi: 10.3389/fmicb.2018.01749 (PMC6085468; doi:10.3389/fmicb.2018.01749)
Supplement: Supplementary Table S2 — Primers used in this study. [file Table_2.DOCX]

**Table S2.** **Primers used in this study^b^**

| **Primer Name** | **Sequence** | **Product Length (bp)** |
| --- | --- | --- |
| **For mutants and promoter reporter plasmid construction** | | |
| D2974LF | GGGAAGCTTCCCCAGCCGGGAAAGATGCCG | 700 |
| D2974LR | GGGTCTAGAGTCGGGCCGCACTCCTGTCTG |  |
| D2974RF | GGGTCTAGACCGATCGGAACGGTGCGCTCG | 791 |
| D2974RR | AAAGGATCCTTCAGGATGCGTGAACGGGTT |  |
| D0556LF | GGGGGATCCCCCACGCTTTTGCTCTGGAAG | 662 |
| D0556LR | GGGTCTAGAGTGGCCGACACGATGACCTCC |  |
| D0556RF | GGGTCTAGATGTTCGCTGTGTCGGCCTGAG | 670 |
| D0556RR | GGGAAGCTTTGGTCCTGGCGTACACAAAAG |  |
| D1193LF | GGGGGATCCGCAAGGTGCTCACCCACGAAC | 701 |
| D1193LR | GGGTCTAGATGGCATGCGCGCAGTGTAGAG |  |
| D1193RF | GGGTCTAGAGCAATGAGCGCACTGTTCGAG | 707 |
| D1193RR | GGGAAGCTTCGTTCGGAGGCGGGGTGAGGA |  |
| D1474LF | GGGGAATTCCACCGCTTCGACGTCGTCGAT | 702 |
| D1474LR | GGGGGATCCATGGGAACGGCGGCGTGGGTG |  |
| D1474RF | GGGGGATCCCCTTTCCCCTTGTGCAACCGG | 700 |
| D1474RR | GGGAAGCTTGGTGCAGGTCGCCCGTCGCCC |  |
| D2566LF | GGGGAATTCCTGACAACGTCGGCGGCGCTG | 705 |
| D2566LR | GGGGGATCCGTCATCGAAGAGGCGCTGCAA |  |
| D2566RF | GGGGGATCCACCATGCAATGAAGACGCTCT | 701 |
| D2566RR | GGGAAGCTTTCAGCAGCGCCGCCAGCAGAC |  |
| D2905LF | GGGGGATCCTGGAAGTCACCTATGCCATCGCCGA | 718 |
| D2905LR | GGGTCTAGAGCCAGGAATGGCACTCATGCGGTCG |  |
| D2905RF | GGGTCTAGATGGCCAAACTCAAGGCATGTCTGG | 669 |
| D2905RR | AAGAAGCTTTGCGCCTTGCCATCGGGGGTG |  |
| D2934LF | GGGGAATTCCATTACCGTCGCTGTAGATGC | 710 |
| D2934LR | GGGGGATCCTATCGAGCATAGCCAGTTCGA |  |
| D2934RF | GGGGGATCCCCGTATGACCCAATCCCTTCC | 699 |
| D2934RR | GGGAAGCTTGCCACGCACGCGTACCTGCCC |  |
| D3099LF | GGGGAATTCCACGCCAACGACCAACCACGT | 714 |
| D3099LR | GGGTCTAGAGTGTCGGCGTGCATCGTCGGA |  |
| D3099RF | GGGTCTAGACTGGGACTGGTGTGACGTGGG | 715 |
| D3099RR | GGGAAGCTTCGGCCAAGCTCGACACCAACG |  |
| D3383LF | GGGGAATTCGCGCTGCACACCGCAACGGCG | 714 |
| D3383LR | GGGTCTAGAGTGCCTGCCGCCAAGCGCCCG |  |
| D3383RF | GGGTCTAGATGAACCGCTCCCACGACCCGG | 703 |
| D3383RR | GGGAAGCTTGCGGCGCTGCTCGGGGCTCAT |  |
| D3864LF | GGGGAATTCGGCGAAGCGCCATTGCTGATG | 704 |
| D3864LR | GGGGGATCCCCATGGGCACCGATCAGAGCA |  |
| D3864RF | GGGGGATCCACGCGCGCGTTTTCGTTTTTA | 700 |
| D3864RR | GGGAAGCTTCGATCCTGCTGCGGCAGACCA |  |
| D2281LF | GGGGAATTCTCGACGCCGACCTGGGCCTGG | 708 |
| D2281LR | GGGTCTAGAGTACTCATGCGGCCACTCCCC |  |
| D2281RF | GGGTCTAGAGATTAATTTTTTCGTGTTAGGAGC | 696 |
| D2281RR | GGGAAGCTTTTTTCGGTCATTTCCACCACG |  |
| D1311LF | GGGGAATTCCTGACCCTGGAGGCCGGCGAA | 701 |
| D1311LR | GGGGGATCCTTCATGCGGACACTAGCTGGA |  |
| D1311RF | GGGGGATCCGACGTTGCAACCACCGCAACG | 685 |
| D1311RR | GGGAAGCTTAACTCCACCGGCGTCTGCAGC |  |
| D2251LF | AAAGGATCCTGCCATGACCGATGTGCTGCG | 690 |
| D2251LR | GGGTCTAGAGCAGAGATGGTCGTCTTCATG |  |
| D2251RF | GGGTCTAGACCCGGGCGGGATAAGGACTACTATG | 714 |
| D2251RR | GGGAAGCTTGGGTATCCAGCGCCCACACGT |  |
| D3843LF | GGGGAATTCCCAGGCGCATGGGCTGTGCTTTT | 698 |
| D3843LR | GGGGGATCCATAGGGCACCATCTTAGCAGT |  |
| D3843RF | GGGGGATCCGCCTGATCCGGTGTGACATTG | 706 |
| D3843RR | GGGAAGCTTTCCTGCGCAGCGGCGGTTTCC |  |
| D3806LF | GGGGAATTCTCCAGGCCAGCGTGTCGGCCA | 750 |
| D3806LR | GGGTCTAGAGAGTGCTCGCGCCACCGGCTT |  |
| D3806RF | GGGTCTAGACGACTGATCGCACAGCGGTCA | 761 |
| D3806RR | GGGAAGCTTATCAGCTGCCCCCAGAGTCGG |  |
| C3843F | GGGGAATTCCAGAACAAGGGCTGGGAAGGC | 1476 |
| C3843R | GGGAAGCTTGACGTCAACACCCAACCGTAT |  |
| C3806F | GGGGAATTCGCGCAGGTGGTCGGGCAGATC | 2471 |
| C3806R | GGGAAGCTTGTTCCTGCGAACCGCAGAGCG |  |
| CC2974F | GGGTCTAGATCCAGTGCCAGGCGTTCGATG | 1121 |
| CC2974R | AAAGGATCCCGCGACTCCTCGGCACTCAGC |  |
| 0742LF | GGGAAGCTTGCGCCTGGCGCCGATGCTGGA | 691 |
| 0742LR | GGGTCTAGACGGCCATCCCTGCGACTCAGCGC |  |
| 0742RF | GGGGGATCCCGCGCGCGCTAGCATCGAGT | 739 |
| 0742RR | GGGGAATTCCGGCTGATTCCTGCCCTGCG |  |
| OE2974F | GGGTCTAGAAATGGCCGAAGTCGATACACC | 622 |
| OE2974R | GGGAAGCTTTCATACACGGTGTCGCTCAC |  |
| Flag2974LF | GGGGAATTCCGATCGTTTGGATCGGTAGGA | 700 |
| Flag2974LR | AAAGGATCCACGGTGTCGCTCACGGGTAGC |  |
| Flag2974RF | GGGTCTAGAGTATGACCAATAACCCTGACATGT | 682 |
| Flag2974RR | AAAAAGCTTGGATGCGTGAACGGGTTTGCT |  |
| 3×Flag-F | GGGGGATCCGATTATAAAGATCATGACGGTGATTATAAAGATCATGACATCGACTACAAGGATGACGATGACAAGCTCGAGTGATCTAGAGGG |  |
| 3×Flag-R | CCCTCTAGATCACTCGAGCTTGTCATCGTCATCCTTGTAGTCGATGTCATGATCTTTATAATCACCGTCATGATCTTTATAATCGGATCCCCC |  |
| GUS3076F | GGGGAATTCCATCCGTACGCCCACGCATCG | 300 |
| GUS3076R | GGGGGTACCCGGAAGACGGTCTGCGTAAGA |  |
| GUS2974F | GGGGAATTCTCCAGTGCCAGGCGTTCGATG | 529 |
| GUS2974R | GGGGGATCCGCGCCCGATCAGGGCGACGAT |  |
|  |  |  |
| **For protein purification** | | |
| E2974F | GGGGGATCCATGGCCGAAGTCGATACACCT | 621 |
| E2974R | GGGGAATTCTCATACACGGTGTCGCTCACG |  |
|  |  |  |
| **For qRT-PCR and sqRT-PCR** | | |
| QRT16s-F | GAGGAAGGTGGGGATGACGTCA | 108 |
| QRT16s-R | GATTGGCTTACCCTCGCGGG |  |
| QRThrpG-F | GCATCCAGGCCCAGCAGATCA | 180 |
| QRThrpG-R | TACCCCAGACTGCCTTGGCCA |  |
| QRThrpX-F | GCGTGGCTGATGTTGAGCCAGC | 180 |
| QRThrpX-R | CTCTTGCGCGCGTTCCAGCGT |  |
| QRThrpA-F | CAACATATACGGCGTGCGTG | 113 |
| QRThrpA-R | CGCTGTTGCCGAAGGTATTG |  |
| QRThrpB-F | ACCTTCGATGCCTGGATTGC | 124 |
| QRThrpB-R | ACAGGCAACACGCGTACAA |  |
| QRThrpC-F | CGTGCAGCATTGGTTGTTCA | 130 |
| QRThrpC-R | GAAGACCAGTTCCTTGGCGA |  |
| QRThrpD-F | ATAGCGGCAGTCTGGAGTTG | 118 |
| QRThrpD-R | CGCAGATCATCGCTCAACAG |  |
| QRThrpE-F | CACACTGCCACTCAGACCTC | 114 |
| QRThrpE-R | TTCGTTCTTCTTCGCGGTCA |  |
| QRThrpF-F | TACGGCAATACGTCGCTCAA | 137 |
| QRThrpF-R | TTGACATTGAACGGAGCCGA |  |
| QRT0241-F | CTTACGACGCACCGCGCTGT | 115 |
| QRT0241-R | CCATGGCTGACGGGTTGCGT |  |
| QRT1553-F | CCGCATATGAACCTGGACGA | 129 |
| QRT1553-R | CGCTGAAGCTTGGTAGGACA |  |
| QRT2974-F | CGAATTGATGCGACAGGAGC | 113 |
| QRT2974-R | CCTCGTAACTCAGCCCTTCC |  |
| RT3591F | GTTGACCGTGGAAGGCGCCAG | 130 |
| RT3591R | GACGTCCATCGCGCCATCGTG |  |
| RT4318F | CGATCCGGCGCAACGCAGTGC | 108 |
| RT4318R | ACGTTGCGCAGCGCCCAGTCG |  |
| RT0241F | CTTACGACGCACCGCGCTGT | 115 |
| RT0241R | CCATGGCTGACGGGTTGCGT |  |
| RT1084F | CGCGCGCTGGTGCCCGACGAG | 137 |
| RT1084R | CCGGGCACCGGCTGCAACACC |  |
| RT1450F | ACTCAGCAATATGCCCGCCAC | 102 |
| RT1450R | AGACCGGAGATCCACAACGCC |  |
| RT2410F | CCGCTGCTTGCTGAGAAGTAT | 115 |
| RT2410R | TACGAAGTGTGGACTGCTGCG |  |
| RT2827F | GCAGGCAATCACGCCAACCGAAGTG | 104 |
| RT2827R | CGCGCGCAATCAGCCCTTTCT |  |
| RT3000F | ATGATTGGCGCCTGGGTCGGG | 109 |
| RT3000R | GCCAGGTGGCACCGATGGTCT |  |
| RT3147F | GGTCAACTGCACCTACTCTCT | 115 |
| RT3147R | GTAAGAAGGGTGCTTGAGAGC |  |
| RT3922F | TCCCGGGGATGCAAGGCGAT | 110 |
| RT3922R | CCACACGGTCCCATCTTTGCA |  |
|  |  |  |
| **For in *vitro* transcription assay** | | |
| ivt3076F | CTGGAGCGGCGGCATGCCAAC | 647 |
| ivt3076R | GCCATCCGGAGCTGCCTGCCC |  |
| ivt3077F | GCGATTCGCCGGTCCACCTG | 582 |
| ivt3077R | CCTCTGGGACCAGAGTCGCCA |  |
|  | | |
| **For ChIP assay** | | |
| hrpAp-F | GATCGGCGAGTCGTTCGTCAT | 480 |
| hrpAp-R | GTTCCCTCTGCGAGGAAGTGA |  |
| hrpBp-F | ACGAAGGGCAGCGTCAGCAAG | 480 |
| hrpBp-R | CTCTTCATTCAATGGCCGTGG |  |
| hrpCp-F | GGGTCGCCCACCGCAAACAGG | 490 |
| hrpCp-R | CTCCGGCCGCTATTGCTGCAC |  |
| hrpDp-F | CGGCAATCGTCGACAGATCGCCCG | 499 |
| hrpDp-R | TCTGCCCAGCTGCGCGCGCAC |  |
| hrpEp-F | GCACACTGCATGTGGAGATCA | 480 |
| hrpEp-R | CTTAGCTGAAGAGAAGTAACG |  |
| hrpFp-F | CCTATGTTACCTCTACCAAGCGACG | 470 |
| hrpFp-R | CCTCGCAGTGACAGAGCAGTG |  |
| hrpXp-F | GCATGCCAACGTGCAGGTGTA | 480 |
| hrpXp-R | CTCTCTGAAGGGGAGGAGCAA |  |
| hrpGp-F | GGGCACACGCTGTTTTCCGGC | 470 |
| hrpGp-R | CGGGCGCAGCCCAGACCATGG |  |
|  |  |  |
| **For EMSA** | | |
| PhrpX-F(FAM) | GCGAGCGATCGCAGATGTTTAG | 354 |
| PhrpX-R(FAM) | CGGAAGACGGTCTGCGTAAGA |  |
| PhrpG-F(FAM) | TTAAAACGCCCCAACCAGCCC | 353 |
| PhrpG-R(FAM) | CGCGTCCTGCGTCAGTACGAA |  |

^b^ The underlined sequences indicate the restriction sites for *Bam*H I, *Eco*R I, *Kpn* I, *Xba* I, and *Hin*d III, respectively.
