# Supplementary material for: Systematic Functional Analysis of Sigma (σ) Factors in the Phytopathogen Xanthomonas campestris Reveals Novel Roles in the Regulation of Virulence and Viability
Source: Front Microbiol. 2018 Aug 3;9:1749. doi: 10.3389/fmicb.2018.01749 (PMC6085468; doi:10.3389/fmicb.2018.01749)
Supplement: Supplementary Table S3 — Statistics analysis of the virulence of Xcc strains. [file Table_3.DOCX]

**Table S3** **Statistics analysis of the virulence of *Xcc* strains**

| Strains | Lesion length (mm) | *P*-values (Student’s *t*-test, compared with the wild type) |
| --- | --- | --- |
|  | Mean ± SD |  |
| Wild type | 11.79 ± 0.73 |  |
| Δ*rpoN1* | 10.92 ± 0.52 | 0.166 |
| Δ*rpoN2* | 11.83 ± 1.19 | 0.958 |
| Δ*rpoN1rpoN2* | 10.66 ± 0.59 | 0.108 |
| Δ*fliA* | 11.06 ± 2.09 | 0.615 |
| Δ*rpoE1* | 8.09 ± 0.96 | 0.007 |
| CΔ*rpoE1* | 10.83 ± 0.98 | 0.253 |
| Δ*rpoE2* | 11.39 ± 0.67 | 0.519 |
| Δ*rpoE3* | 11.21 ± 0.74 | 0.388 |
| Δ*rpoE4* | 12.17 ± 1.53 | 0.726 |
| Δ*rpoE5* | 11.39 ± 0.64 | 0.515 |
| Δ*rpoE6* | 11.05 ± 1.52 | 0.505 |
| Δ*rpoE7* | 11.09 ± 1.74 | 0.570 |
| Δ*rpoE8* | 10.92 ± 0.93 | 0.276 |
| Δ*rpoE9* | 11.14 ± 0.37 | 0.266 |
| Δ*rpoE10* | 10.78 ± 0.89 | 0.206 |
| Δ9 | 11.28 ± 0.67 | 0.423 |
| Δ10 | 7.95 ± 0.60 | 0.002 |
| Δ11 | 8.00 ± 0.84 | 0.004 |
